# Comparative genomics, infectivity and cytopathogenicity of Zika viruses produced by acutely and persistently infected human hematopoietic cell lines

**DOI:** 10.1371/journal.pone.0203331

**Published:** 2018-09-07

**Authors:** Bingjie Li, Hsiao-Mei Liao, Hebing Liu, Shien Tsai, Jing Zhang, Guo-Chiuan Hung, Pei-Ju Chin, Yamei Gao, Shyh-Ching Lo

**Affiliations:** 1 Tissue Microbiology Laboratory, Division of Cellular and Gene Therapies, Office of Tissues and Advanced Therapies, Center for Biologics Evaluation and Research, Food and Drug Administration, Silver Spring, Maryland, United States of America; 2 Lab of Pediatric and Respiratory Viral Diseases, Division of Viral Products, Office of Vaccines Research and Review, Center for Biologics Evaluation and Research, Food and Drug Administration, Silver Spring, Maryland, United States of America; University of Minnesota College of Veterinary Medicine, UNITED STATES

## Abstract

Zika virus (ZIKV), an arthropod-borne virus, has emerged as a major human pathogen. Prolonged or persistent ZIKV infection of human cells and tissues may serve as a reservoir for the virus and present serious challenges to the safety of public health. Human hematopoietic cell lines with different developmental properties revealed differences in susceptibility and outcomes to ZIKV infection. In three separate studies involving the prototypic MR 766 ZIKV strain and the human monocytic leukemia U937 cell line, ZIKV initially developed only a low-grade infection at a slow rate. After continuous culture for several months, persistently ZIKV-infected cell lines were observed with most, if not all, cells testing positive for ZIKV antigen. The infected cultures produced ZIKV RNA (v-RNA) and infectious ZIKVs persistently (“persistent ZIKVs”) with distinct infectivity and pathogenicity when tested using various kinds of host cells. When the genomes of ZIKVs from the three persistently infected cell lines were compared with the genome of the prototypic MR 766 ZIKV strain, distinct sets of mutations specific to each cell line were found. Significantly, all three “persistent ZIKVs” were capable of infecting fresh U937 cells with high efficiency at rapid rates, resulting in the development of a new set of persistently ZIKV-infected U937 cell lines. The genomes of ZIKVs from the new set of persistently ZIKV-infected U937 cell lines were further analyzed for their different mutations. The 2^nd^ generation of persistent ZIKVs continued to possess most of the distinct sets of mutations specific to the respective 1^st^ generation of persistent ZIKVs. We anticipate that the study will contribute to the understanding of the fundamental biology of adaptive mutations and selection during viral persistence. The persistently ZIKV-infected human cell lines that we developed will also be useful to investigate critical molecular pathways of ZIKV persistence and to study drugs or countermeasures against ZIKV infections and transmission.

## Introduction

Transmission of ZIKV in French Polynesia and South America has been associated with the development of pathologic neurological symptoms and central nervous system abnormalities in the fetus [1–4]. Studies on the newly emerging infection of ZIKV have rapidly progressed on various fronts, including development of methods for rapid virus detection, medical treatments and vaccines against the Zika flavivirus [5–10]. Earlier studies revealed that ZIKV could be found in urine, semen, saliva or tears of infected patients long after the virus could no longer be detected in the patients’ blood [11–14]. Long-term presence of v-RNA in body fluids is an indicator for prolonged or persistent viral infection in the body. However, cells that support continuous ZIKV replication and mechanisms by which the virus establishes persistence in various human tissues remain enigmatic.

Persistently flavivirus-infected cells hidden in tissues are often difficult to detect and may lead to devastating medical consequences. The incidents of transmitting fatal infection of West Nile Virus (WNV), another flavivirus, from WNV-infected organ donors who were no longer viremic during blood testing to recipients through organ transplantation are well-documented [15, 16]. To effectively prevent ZIKV transmission through transfusion of infected blood and particularly, through transplantation of infected organs or tissues, we need a better appreciation of the cells harboring the infectious virus and serving continuously as a ZIKV reservoir for a prolonged period of time. In addition, a better understanding of the key mechanisms of developing ZIKV persistence in these cells is needed.

An earlier study of acute experimental ZIKV infection in Rhesus Macaques revealed that the detection of v-RNA was more persistent in whole blood compared to plasma. Moreover, the study showed that many tissues contained v-RNA 14 days post-infection, when the infected animals were no longer viremia, with the highest levels in hemato-lymphatic tissues, such as lymph nodes and spleen [17]. Two other studies involving experimentally ZIKV-infected rhesus monkeys also reported that lymphoid tissues and central nervous system (CNS) cells had prolonged viral persistence [18, 19]. Thus, all three non-human primate studies of ZIKV infections demonstrated that the hemato-lymphatic system had the most prolonged infection [17–19]. Consistent with the study results of ZIKV-infected monkeys, the study of patients who had benign ZIKV infections similarly revealed that ZIKV v-RNA persisted in whole blood samples substantially longer than in plasma [20]. Taken together, these findings suggested the presence of yet-to-define cells allowing prolonged or persistent ZIKV infection in the hemato-lymphatic tissues and blood of the ZIKV-infected experimental monkeys as well as patients.

To investigate possible ZIKV persistence in cells of hemato-lymphatic system and blood, we examined the relative susceptibility of human hematopoietic cell lines with different developmental characteristics to infection of the prototype MR 766 ZIKV strain and focused on the duration as well as the outcome(s) of the infection. Significantly, we found that human monocytic leukemia/histiocytic lymphoma-originated U937 cell line [21], initially showing a slow rate and low grade infection using ZIKV MR 766 strain, could develop into persistently ZIKV-infected cell lines in which most of the cells in the culture were positive for ZIKV antigens despite no apparent cytopathological changes. We measured the amounts of v-RNA genomes and compared the titers of infectious ZIKV virions produced by persistently ZIKV-infected U937 cells growing as continuous cultures established in the context of 3 separate experiments. We first compared the “persistent ZIKVs” produced by the 3 persistently ZIKV-infected cell lines with the inoculum prototype strain MR 766 ZIKV for their infectivity and pathogenicity against human hematopoietic cells with different developmental characteristics. Furthermore, we conducted a detailed comparative genome sequence analyses among 1) the inoculum prototype strain MR 766 ZIKV, 2) the “early phase ZIKVs” produced by U937 cells infected by MR 766 ZIKV in the first 1–2 weeks, 3) the “persistent ZIKVs” (1^st^ generation) produced by the 3 continuous U937 cell lines that developed viral persistence following infections of prototype strain MR 766 ZIKV, and 4) the “persistent ZIKVs” (2^nd^ generation) produced by the 3 continuous U937 cell lines that developed viral persistence following infections with the 3 respective 1^st^ generation persistent ZIKVs. The apparent dynamic interplay between ZIKVs and the infected host cells to confer viral persistence following each viral infection is discussed.

## Results

### Susceptibility of immortalized human hematopoietic cells with different developmental characteristics to ZIKV infection

A number of human B cell lines, T cell lines and myelogenous/monocytic cell lines (see Table 1) were infected by ZIKV at MOI of 2 for 2 hours, washed twice using PBS and re-suspended in fresh culture media. The effectiveness and efficiency of ZIKV infection in the cultured human hematopoietic cells were monitored by the kinetics of cells becoming positive for ZIKV-specific antigens using an immunofluorescent assay (IFA) and developing virus infection associated cytopathological effect (CPE) changes with cytolytic necrosis. Cells of non-ZIKV-infected control cultures were studied in parallel for comparison.

**Table 1:** Human hematopoietic cells with different developmental characteristics showed different susceptibility to ZIKV (strain MR 766) infection.

Name of human cell line	Cell type	Susceptibility to infection*	Viral CPE observed**	v-RNA genome copies/ml (Day 6)
**B cell lines**				
CCRF-SB	B lymphoblastoid cells	Susceptible	Y	1.60×10^8^
RPMI 8392	B lymphoblastoid cells	Susceptible	Y	1.52×10^8^
K2267-Mi	B lymphoblastoid cells	Susceptible	Y	2.18×10^8^
824–00	B lymphoblastoid cells	Susceptible	Y	8.07×10^8^
4695EB	B lymphoblastoid cells	Susceptible	Y	2.60×10^9^
**T cell lines**				
CCRF-CEM	T lymphoblastoid cells	Resistant	N	NA
CCRF-HSB2	T lymphoblastoid cells	Resistant	N	NA
Molt-3	T lymphoblastoid cells	Resistant	N	NA
RPMI 8402	T lymphoblastoid cells	Resistant	N	NA
**Myelogenous Monocytic cell lines**				
K562 (K)	Chronic myelogenous leukemia	Susceptible	Y	1.02×10^10^
U937 (U)	Histiocytic lymphoma	Susceptible^#^	N	2.19×10^7^
THP-1	Monocytic leukemia	Resistant	N	NA
HL-60	Promyelocytic leukemia	Resistant	N	NA
KG-1	Myelocytic leukemia	Resistant	N	NA

*Susceptibility to infection: Finding ZIKV-specific antigen positive cells by immunofluorescent staining within a week of ZIKV infection in the culture.

**#Susceptible:** ZIKV-specific antigen positive cells could be found in the culture, but only at low numbers.

**CPE observed: Cells showing cytopathological changes and cytolytic necrosis that resulted in significantly lower numbers (<50%) of viable cells found in the ZIKV-infected cultures in comparison with those in the non-ZIKV-infected control cultures.

**Fig 1:**
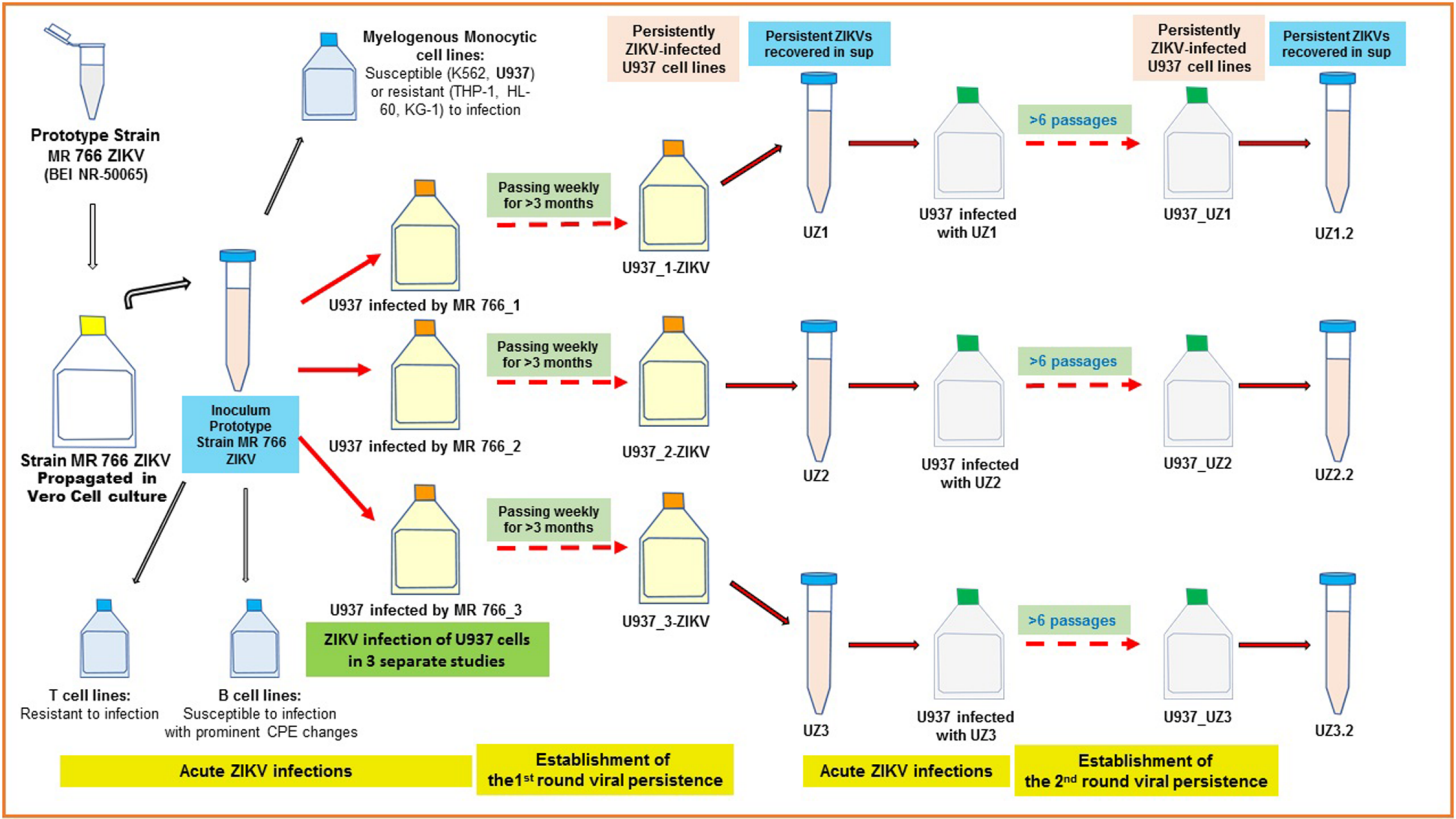
The schematic illustration of the experimental workflow and the establishment of persistently ZIKV-infected U937 cell lines.

Table 1 summarizes the study results showing that all five examined B lymphoblastoid cell lines were highly susceptible to ZIKV infection, turning positive for ZIKV antigens in IFA within the first few days. These B lymphoblastoid cell lines also developed prominent CPE changes with cell necrosis or cytolysis in the cultures observed within a week following ZIKV infection. ZIKV v-RNA genomes (>10^8^ copies/ml) were found in the culture supernatants of these human B cell lines. In contrast, T lymphoblastoid cell lines appeared to be very resistant to the infection of ZIKV. None of the four T lymphoblastoid cell lines turned positive for ZIKV-specific antigens in IFA or developed detectable CPE changes following more than 1 week of ZIKV infection in the cultures. The study revealed ZIKV infectivity of human myelogenous/monocytic cell lines varied. The three myelogenous cell lines, THP-1, HL-60 and KG-1, were resistant to the ZIKV infection without having turned into ZIKV-specific antigens positive cells or having developed CPE changes with cell necrosis in the infected cultures. However, the prototype MR 766 ZIKV strain could infect 2 of the human myeloid cell lines, K562 and U937. Fig 1 provides a schematic diagram that depicts the workflow of studying ZIKV infections in different human hematopoietic cell lines and the process of establishing persistently ZIKV-infected human monocytic/histiocytic U937 cell lines.

### Prototype strain MR 766 ZIKV infected myelogenous K562 cell line and monocytic/histiocytic U937 cell line at different rates with different efficiencies

**Fig 2:**
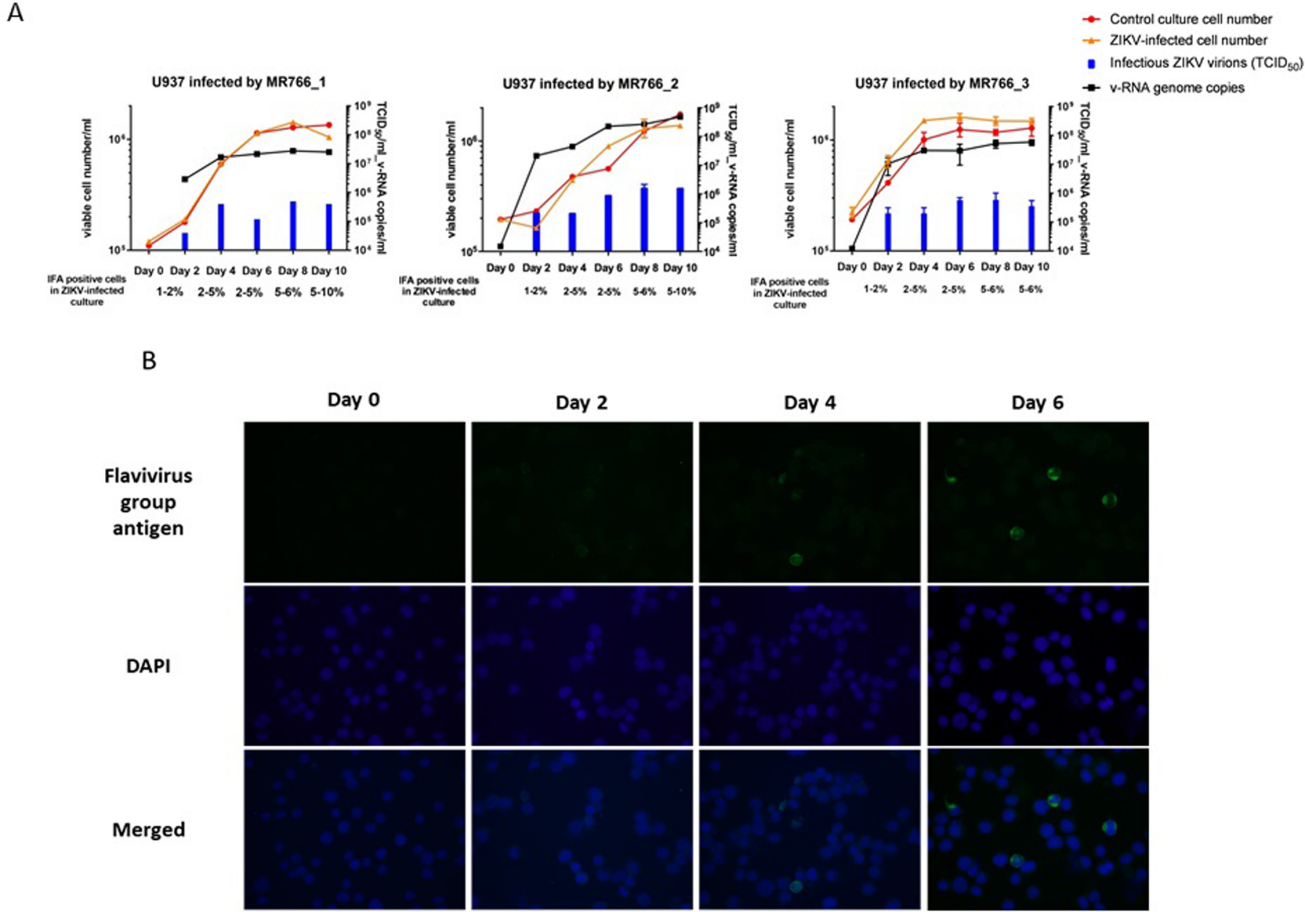
**(A) Cell growth, numbers of cells turning IFA-positive for ZIKV antigen, production of ZIKV RNA genomes and infectious virions in U937 cell cultures with and without infection of Prototype ZIKV Strain MR 766 in 3 separate studies.** Viable cells were determined by trypan blue dye exclusion. Quantitation of v-RNA ZIKV genomes by RT-PCR and titration of infectious ZIKV virions by TCID_50_ assay were examined in the supernatants of U937 cell cultures every 2 days. No ZIKV v-RNA and no infectious ZIKV virion could be detected in supernatants of the 3 non-ZIKV infected control U937 cell cultures. **(B) IFA of cells positively producing ZIKV-specific antigens in U937 cell culture infected with ZIKV Strain MR 766 in the early phase.** Anti-flavivirus group antigen monoclonal antibody was used as the primary IFA antibody for staining day 0 to day 6 cells in the U937-MR 766_1 culture. Merged: the merged image stacks the immunofluorescent staining of flavivirus group antigen and DAPI nuclear staining of cells.

The human cell line K562 that originated from myelogenous leukemia was highly susceptible to ZIKV infection. Specifically, more than 70% of myelogenous K562 cells were already turning positive for immunofluorescent staining of ZIKV antigens in the culture 3 days post ZIKV infection. Many K562 cells had prominent CPE changes and underwent cytolytic necrosis resulting in significantly lower number (~30%) of viable cells than that in the non-infected control culture at day 4. A large amount of ZIKV v-RNA genomes (up to 10^10^ copies/ml) were found in the culture supernatant (Table 1). In comparison, the monocytic leukemia/histiocytic lymphoma-originated U937 cells were much less susceptible to ZIKV infection. Fig 2A shows consistent slow rates and low grade of ZIKV infections in the U937 cell cultures infected with the prototype strain MR 766 ZIKV in 3 separate studies (U937-MR 766_1, U937-MR 766_2 and U937-MR 766_3). Only small numbers of cells tested IFA-positive for ZIKV specific antigen in the cultures following 1–2 weeks of ZIKV infections (Fig 2B). Moreover, the prominent CPE changes and cell loss seen in the ZIKV-infected culture of myelogenous K562 cells could not be detected in the monocytic/histiocytic cultures of U937 cells with the low grade ZIKV infection. In all 3 experiments involving U937 cells, the numbers of viable U937 cells found daily in the ZIKV-infected cultures and the non-infected control cultures were essentially the same.

### Development of persistently ZIKV-infected U937 cell lines

**Fig 3:**
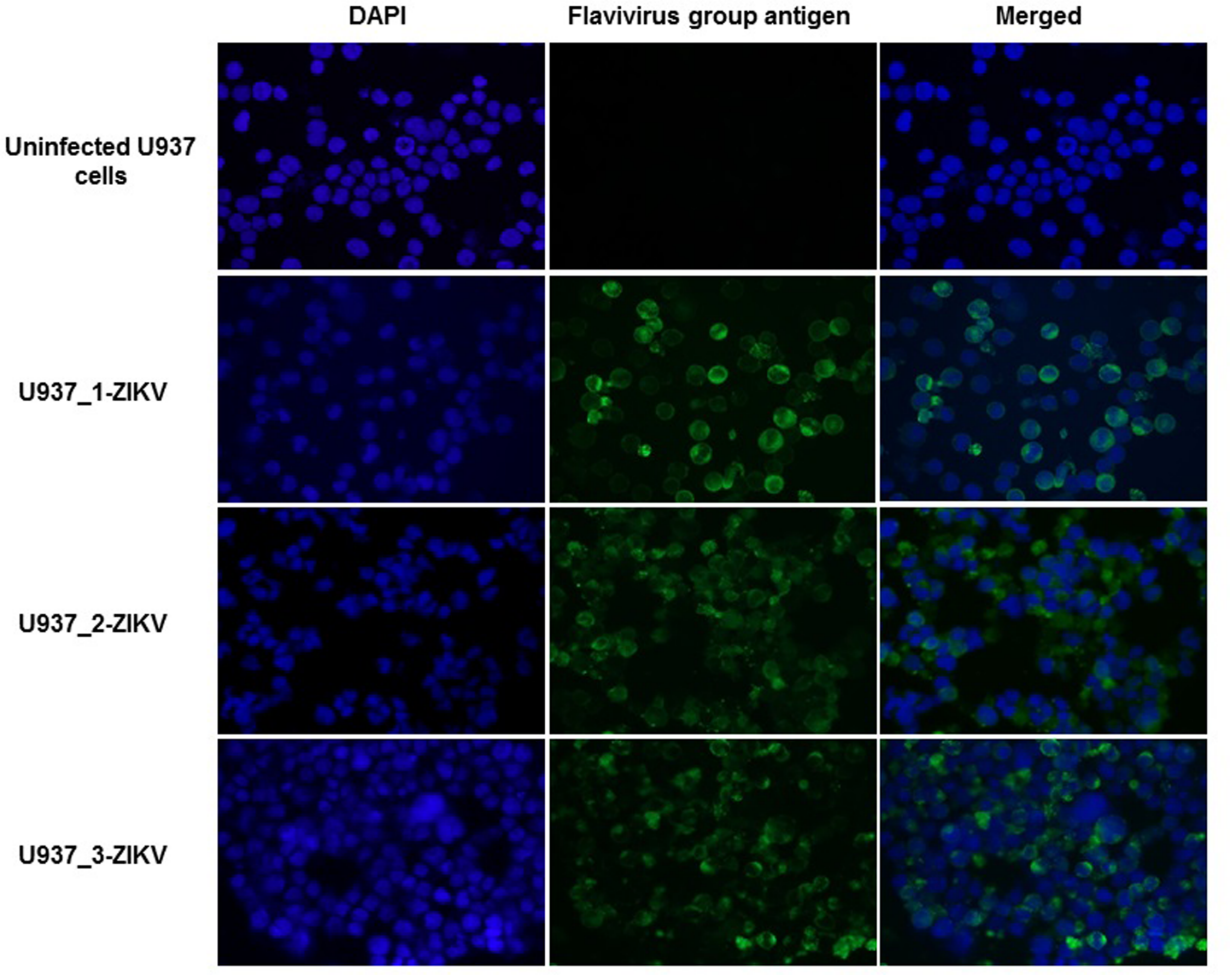
IFA of ZIKV antigen-positive cells in the U937 cell cultures continuously passed for more than 2 months following infections of Prototype ZIKV Strain MR 766. The 3 continuous U937 cell lines U937_1-ZIKV (A), U937_2-ZIKV (B) and U937_3-ZIKV (C) appeared to be persistently infected with ZIKV. Most of cells in the 3 cultures were positively stained for ZIKV specific antigen. Merged: the merged image stacks the immunofluorescent staining of flavivirus group antigen and DAPI nuclear staining of cells.

Although ZIKV infection in the U937 cell cultures had only small numbers of ZIKV antigen positive cells and essentially no cells dying of viral infection-associated CPE in the first 1–2 weeks, both ZIKV v-RNA genomes (~4×10^7^ copies/ml) and infectious virions (~5×10^5^ ID_50_ units/ml) could be detected in supernatants of the 3 ZIKV-infected cultures (Fig 2A). Noticeably, increasing numbers of U937 cells were becoming IFA-positive for ZIKV antigen in the cultures that were diluted and replenished weekly with fresh medium to support continued growth of the viable cells in cultures. By continuously passing U937 cells that were growing in the MR 766 strain ZIKV-infected cultures established separately in the 3 studies U937-MR766_1, U937-MR766_2 and U937-MR766_3 for more than 3 months, we found most, if not all, cells in the 3 cultures tested positive for ZIKV-specific antigen (Fig 3). The continuous U937 cell lines with apparent persistent ZIKV infection established from the 3 separate experiments were designated as U937_1-ZIKV, U937_2-ZIKV and U937_3-ZIKV, respectively (Fig 1). Transmission electron microscopy (TEM) examination of the persistently ZIKV-infected U937 cells revealed large numbers of electron-dense particles with size of ~35 nm in the cytoplasm. Although some of these “virus-like” particles were found in the vesicles, most of them appeared to be free of association with any subcellular structures in the cytoplasm. The nature of these particles in the cytoplasm needs further investigations (**S1 Fig**).

### ZIKV v-RNA genomes and infectious virions produced by the persistently ZIKV-infected U937 cell lines

**Fig 4:**
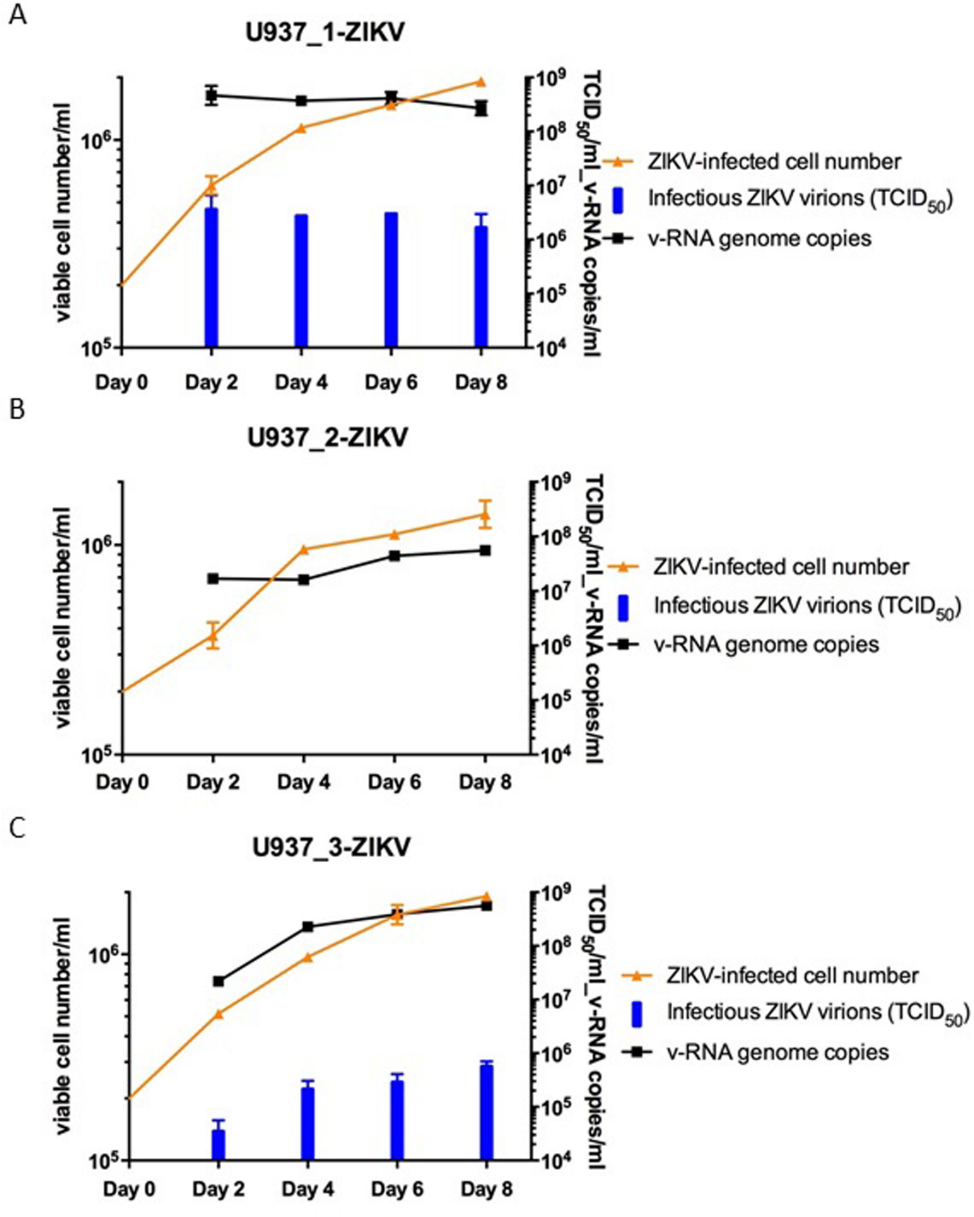
Cell growth, productions of ZIKV RNA genomes and infectious virions in cultures of the 3 continuous U937 cell lines that developed into viral persistence following Prototype ZIKV Strain MR 766 infection. Cells in the persistently ZIKV-infected cultures U937_1-ZIKV (A), U937_2-ZIKV (B) and U937_3-ZIKV (C), were harvested by centrifugation and re-suspended in fresh culture media every 2 days. The amounts of ZIKV RNA genomes and infectious virions produced into supernatants of the cultures in 2 days were quantified by qPCR and titrated by TCID_50_ assay against Vero cells.

We measured both ZIKV v-RNA genomes and infectious virions produced by U937 cells after establishment of persistent ZIKV infection in the 3 ZIKV-infected cultures. In this study, U937 cells in suspension of each persistently ZIKV-infected culture were harvested by centrifugation and re-suspended in fresh media every 2 days. We quantitatively measured both the copy numbers of ZIKV v-RNA genome by RT-PCR and the titers of ZIKV virions that were capable of infecting Vero cells (TCID_50_ units), released into the culture supernatants in 2 days (Fig 4). Panel A graph in Fig 4 shows that ~4×10^8^ copies/ml of ZIKV genome RNA and ~3×10^6^ ID_50_ units/ml of infectious ZIKV virions were consistently produced and released into culture supernatants by 0.5–1×10^6^ cells/ml of the cultured U937_1-ZIKV cell line in 2 days. The ratios between copy numbers of ZIKV v-RNA genome and titers of infectious virions in the culture supernatants were ~100-fold.

Panel B graph in Fig 4 shows that ~6 ×10^7^ copies/ml of ZIKV v-RNA genome were produced and released into culture supernatants by 0.5–1×10^6^ cells/ml of the cultured U937_2-ZIKV cell line in 2 days. However, infectious of ZIKV virions could not be documented in the culture supernatants using the standard TCID_50_ assay. No typical CPE changes with associated cytolytic necrosis and cell sloughing off in the wells were detected in the TCID_50_ assay conducted on Vero cells. However, occasional foci of cell aggregates with atypical CPE were seen in the wells seeded with Vero cells and inoculated with low dilution (≤10^2^) of the supernatants obtained from the culture of U937_2-ZIKV cell line (S2 Fig). The U937_2-ZIKV culture could be producing ≤10^2^ ID_50_ unit/ml of infectious ZIKV virions in the supernatant, if the atypical CPE without cytolytic necrosis or cell sloughing was considered as positive of ZIKV infection in the TCID_50_ assay against cultured Vero cells, The ratios between copy numbers of ZIKV v-RNA genome and titers of infectious ZIKV virions found in the culture supernatants appeared to be extremely high (near 10^6^ fold). Panel C graph in Fig 4 shows that ~6×10^8^ copies/ml of ZIKV v-RNA genomes and 6–8×10^5^ ID_50_ units/ml of infectious virions were consistently produced and released into culture supernatants by 0.5–1×10^6^ cells/ml of the cultured U937_3-ZIKV cell line in 2 days (Fig 4C). The ratios between copy numbers of ZIKV v-RNA genome and titers of infectious ZIKV virions found in the culture supernatants were ~1000-fold.

### Susceptibility of human hematopoietic cells with different developmental characteristics to infections of ZIKVs produced by the persistently ZIKV- infected U937 cell lines

We examined the cell infection specificity, efficiency and pathogenicity of the ZIKVs produced by the U937 cell lines after establishment of ZIKV persistence in comparison with those of the prototype MR 766 strain ZIKV. Like the prototype ZIKV Strain MR 766, the “persistent ZIKVs” (UZ1, UZ2 and UZ3) produced in the 3 cultures of persistently ZIKV-infected U937 cell lines **(**U937_1-ZIKV, U937_2-ZIKV and U937_3-ZIKV, Fig 1) could not infect the examined human T cell lines (Table 2). However, the 3 “persistent ZIKVs” also could not infect the human B cell lines CCRF-SB and RPMI8392 that were susceptible to infection by the prototype strain MR 766 ZIKV. Moreover, although the persistent ZIKVs UZ1 and UZ3 could infect human B cell lines K2267-Mi, 824–00 and 4695EB and effectively turn them IFA-positive for ZIKV-specific antigen, they did not produce prominent CPE changes with associated cytolytic necrosis found in the strain MR 766 ZIKV-infected cultures of these B cell lines (Table 2). The study further revealed that the persistent ZIKV UZ2 that exhibited very low infectivity and cyto- pathogenicity in the TCID_50_ assay against Vero cells (Fig 4, graph B) could not infect any of the 5 human B cell lines that were highly susceptible to infection by the prototype strain MR 766 ZIKV. However, like the prototype strain MR 766 ZIKV, all 3 persistent ZIKVs, UZ1, UZ2 and UZ3, could effectively infect human myeloid hematopoietic K562 cells (Table 2). Nearly all K562 cells in the cultures infected by the 3 persistent ZIKVs became IFA-positive for ZIKV antigen in less than a week. The K562 cells in the infected-cultures showed prominent CPE changes and had extensive cytolytic necrosis **(S3 Fig)**. Hence, it was difficult to maintain continued growth of the remaining viable K562 cells in the cultures infected by the 3 persistent ZIKVs after a week. Like the persistent ZIKV UZ2, ZIKV virions released in the culture of UZ2-infected K562 cell exhibited no infectivity with clear cytolytic necrosis and cell sloughing in the TCID_50_ assay using Vero cells (Panel B graph in S3 Fig).

**Table 2:** Susceptibility of human hematopoietic cell lines to infection of ZIKV MR 766 and the persistent ZIKVs UZ1, UZ2 and UZ3.

Inoculated ZIKV	None		MR 766		UZ1		UZ2		UZ3	
v-RNA genome copy number of inoculation	None		2 × 10^8^		2 × 10^8^		2×10^8^		2 × 10^8^	
**IFA Observation**	**Day 5**	**Day 7**	**Day 5**	**Day 7**	**Day 5**	**Day 7**	**Day 5**	**Day 7**	**Day 5**	**Day 7**
**B cell lines**										
**CCRF-SB**	N	N	>30%+	70% +, CPE	N	N	N	N	N	N
**RPMI 8392**	N	N	100%+, CPE	100% +, CPE	N	N	N	N	N	N
**K2267-Mi**	N	N	10%+	100% +, CPE	~10%+	100% +	N	N	10%+	50%+
**824–00**	N	N	>30%+	100% +, CPE	>90%+	100% +	N	N	50%+	99%+
**4695EB**	N	N	100%+, CPE	100% +, CPE	>90%+	100% +	N	N	70%+	100%+
**T cell lines**										
**CCRF-CEM**	N	N	N	N	N	N	N	N	N	N
**CCRF-HSB2**	N	N	N	N	N	N	N	N	N	N
**Molt-3**	N	N	N	N	N	N	N	N	N	N
**RPMI 8402**	N	N	N	N	N	N	N	N	N	N
**Myelogenous Monocytic cell lines**										
**K562 (K)**	N	N	>90%+, CPE*	100% +, CPE*	100%+, CPE*	100%+, CPE*	100%+, CPE*	100% +, CPE*	100%+, CPE*	100%+, CPE*
**U937 (U)**	N	N	1–2%+	2–5% +	100%+, CPE*	100%+, CPE*	100%+, CPE*	100% +, CPE*	100%+, CPE*	100%+, CPE*
**THP-1**	N	N	N	N	N	N	N	N	N	N
**HL-60**	N	N	N	N	N	N	N	N	N	N
**KG-1**	N	N	N	N	N	N	N	N	N	N

All testing cultures were set up under 10 ml volume with 2 × 10^5^ cells/ml. The testing cultures were inoculated with the supernatants prepared from cultures of the prototype ZIKV MR 766-infected Vero cells or the persistently ZIKV-infected U937 cells (U937_UZ1, U937_UZ2 and U937_UZ3) at MOI of 1 for ZIKV MR 766 (with 2 ×10^7^ MR 766 v-RNA genome copies/ml) or with the same amount of ZIKV v-RNA genomes for UZ1, UZ2 and UZ3.

The percentage of IFA positive cells was estimated under fluorescent microscope at 200x and 400x.

CPE*: CPE with extensive cytolytic changes and cell death.

**Fig 5:**
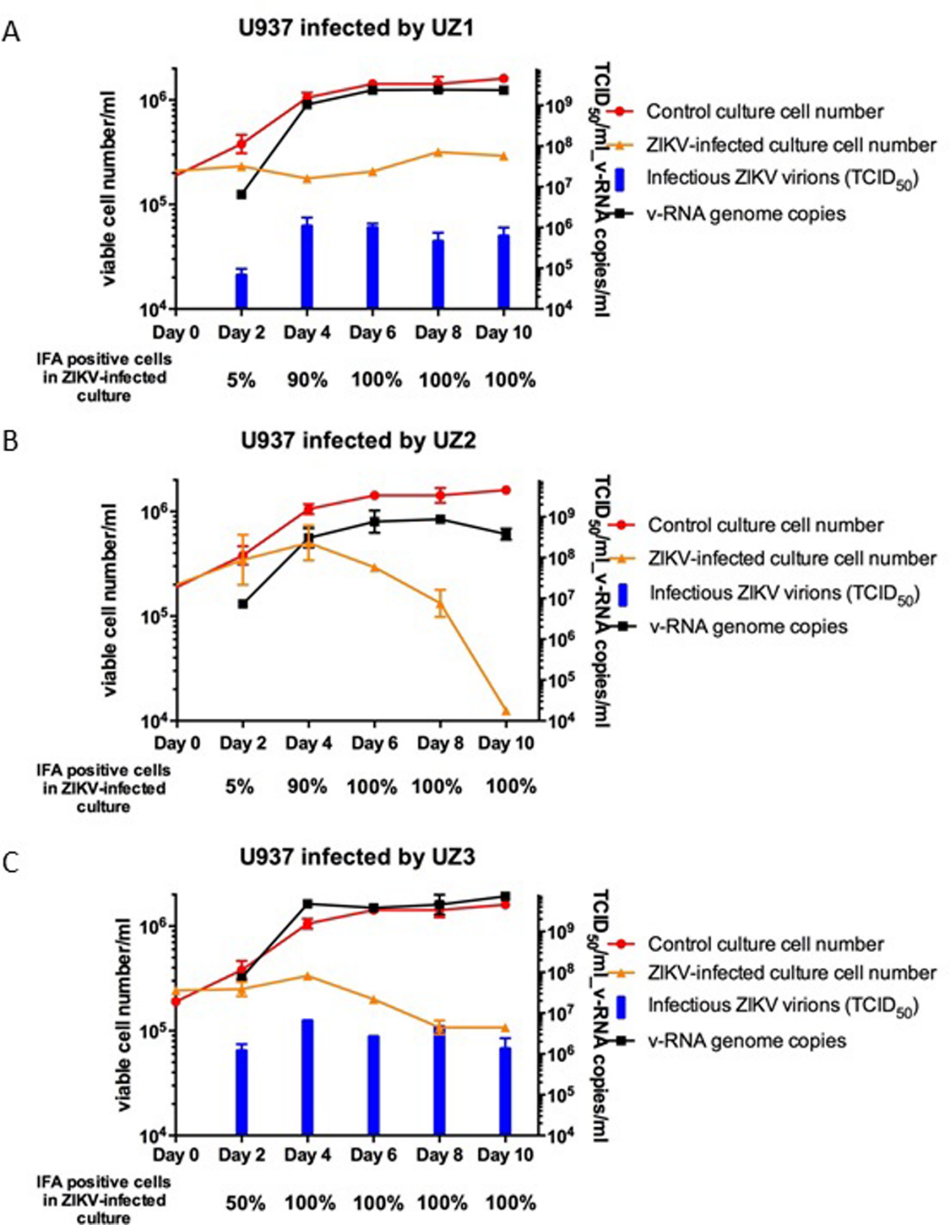
**Cell growth, numbers of cells IFA-positive for ZIKV antigen, productions of ZIKV RNA genomes and infectious virions in cultures of fresh U937 cells infected with the 3 persistent ZIKVs UZ1(A), UZ2 (B) and UZ3 (C).** The cultures of fresh U937 cells (2×10^5^ cells/ml) were infected with the 3 persistent ZIKVs (~10^7^ copies of ZIKV v-RNA genome/ml) prepared from the culture supernatants of persistently ZIKV-infected U937 cell lines U937_1-ZIKV, U937_2-ZIKV and U937_3-ZIKV. Prominent CPE with extensive cytolysis and cell loss were seen in all the 3 cultures infected with the 3 persistent ZIKVs UZ1, UZ2 and UZ3. The amounts of ZIKV RNA genomes and infectious virions produced into supernatants of the ZIKV-infected cultures were quantified by qPCR and titrated by TCID_50_ assay against Vero cells.

In contrast to the prototype strain MR 766 ZIKV; all 3 established “persistent ZIKVs” could rapidly infect fresh U937 cells in culture with high efficiency (Table 2). Fig 5 shows that more than 90% of U937 cells were positive for ZIKV antigen in the cultures of infected by each persistent ZIKVs at day 4, when <5% of U937 cells in the cultures infected by prototype MR 766 ZIKV strain tested positive (Fig 2). Furthermore, infections of the 3 persistent ZIKVs in the U937 cell cultures produced prominent CPE changes with cytolytic necrosis, not observed in the U937 cell cultures infected by the prototype strain MR 766 ZIKV. The number of viable cells in the U937 cell cultures infected by each of the 3 persistent ZIKVs after a week were significantly lower (<20%) than that in the non-infected control culture (Fig 5). The persistent ZIKV UZ2 that did not effectively infect Vero cells and all the human B cell lines appeared to be the most cytopathogenic ZIKV that produced the most serious CPE and cytolytic necrosis in the infected U937 cells culture (Panel B graph in Fig 5). Like the persistent ZIKV UZ2, ZIKV virions produced in the culture of UZ2-infected U937 cells showed no clear infectivity with detectable cytolytic necrosis in the TCID_50_ assay using Vero cells.

### Establishing a 2^nd^ generation of persistently ZIKV-infected U937 cell lines from fresh U937 cell cultures infected with the 1^st^ generation persistent ZIKVs

**Fig 6:**
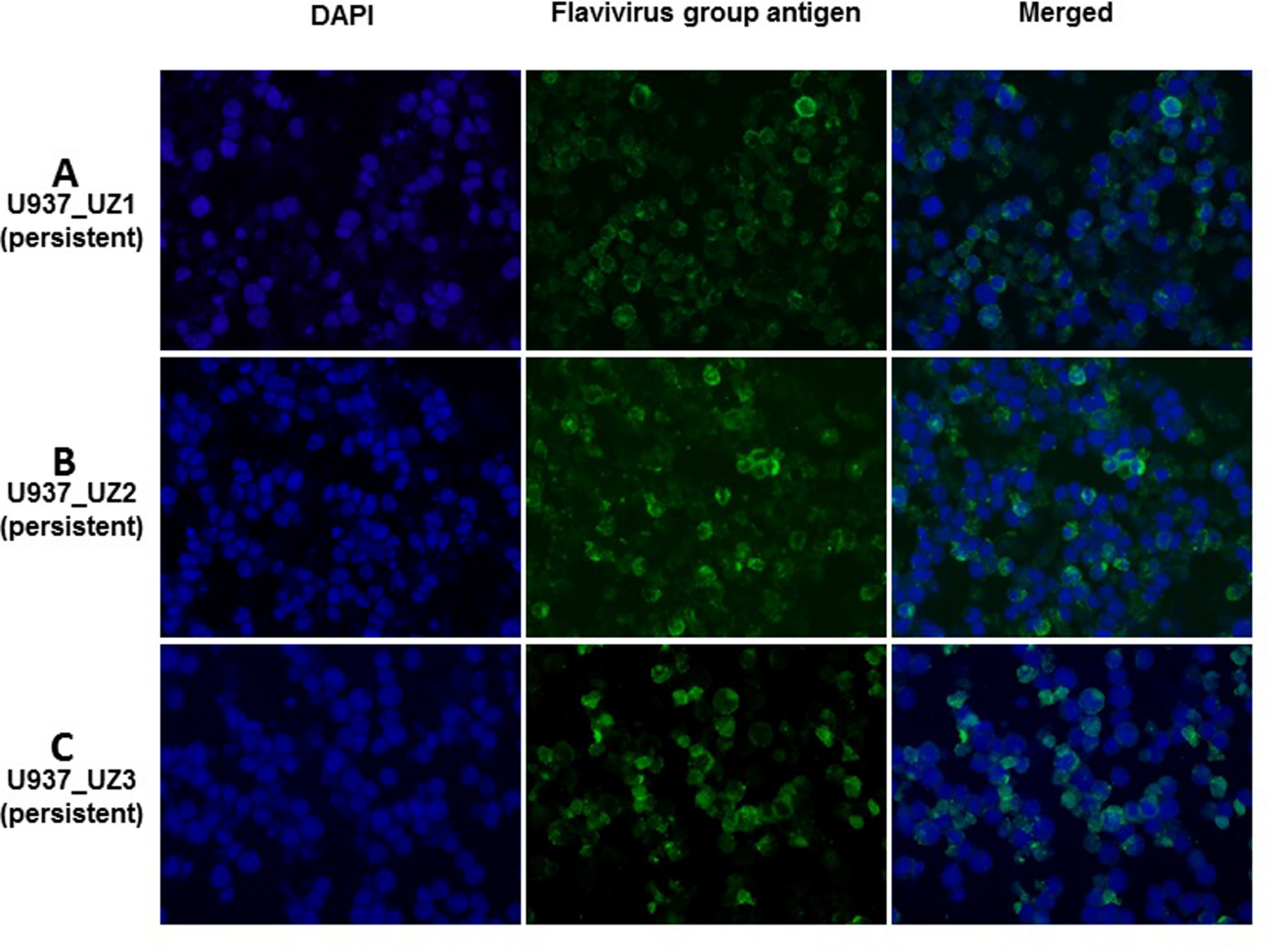
IFA of ZIKV antigen-positive cells in the 3 continuous U937 cell lines established from continuously passing the viable cells that recovered from U937 cell cultures infected with the 3 persistent ZIKVs UZ1, UZ2 and UZ3. The 3 continuous U937 cell lines were persistently infected with ZIKV and designated as (A) U937_UZ1 (persistent), (B) U937_UZ2 (persistent) and (C) U937_UZ3 (persistent), respectively. Merged: the merged image stacks the immunofluorescent staining of flavivirus group antigen and DAPI nuclear staining of cells.

The small numbers of viable U937 cells began to re-grow in the UZ1 and UZ3-infected cultures after 2 weeks. An even smaller number of viable U937 cells in the UZ2-infected culture also recovered and began to re-grow after 3 weeks. By further passing the U937 cells that were growing in the cultures infected by the respective 3 persistent ZIKVs for more than 2 months (more than 6 passages), we established 3 continuous U937 cell lines with most, if not all, of the growing cells in the cultures being IFA-positive for ZIKV-specific antigen (Fig 6). These U937 cell lines with apparent persistent ZIKV infection (the 2^nd^ generation) established by infecting fresh U937 cells in culture with the 3-pre-established persistent ZIKVs UZ1, UZ2 and UZ3 were designated as U937_UZ1, U937_UZ2 and U937_UZ3, respectively (Fig 1).

**Fig 7:**
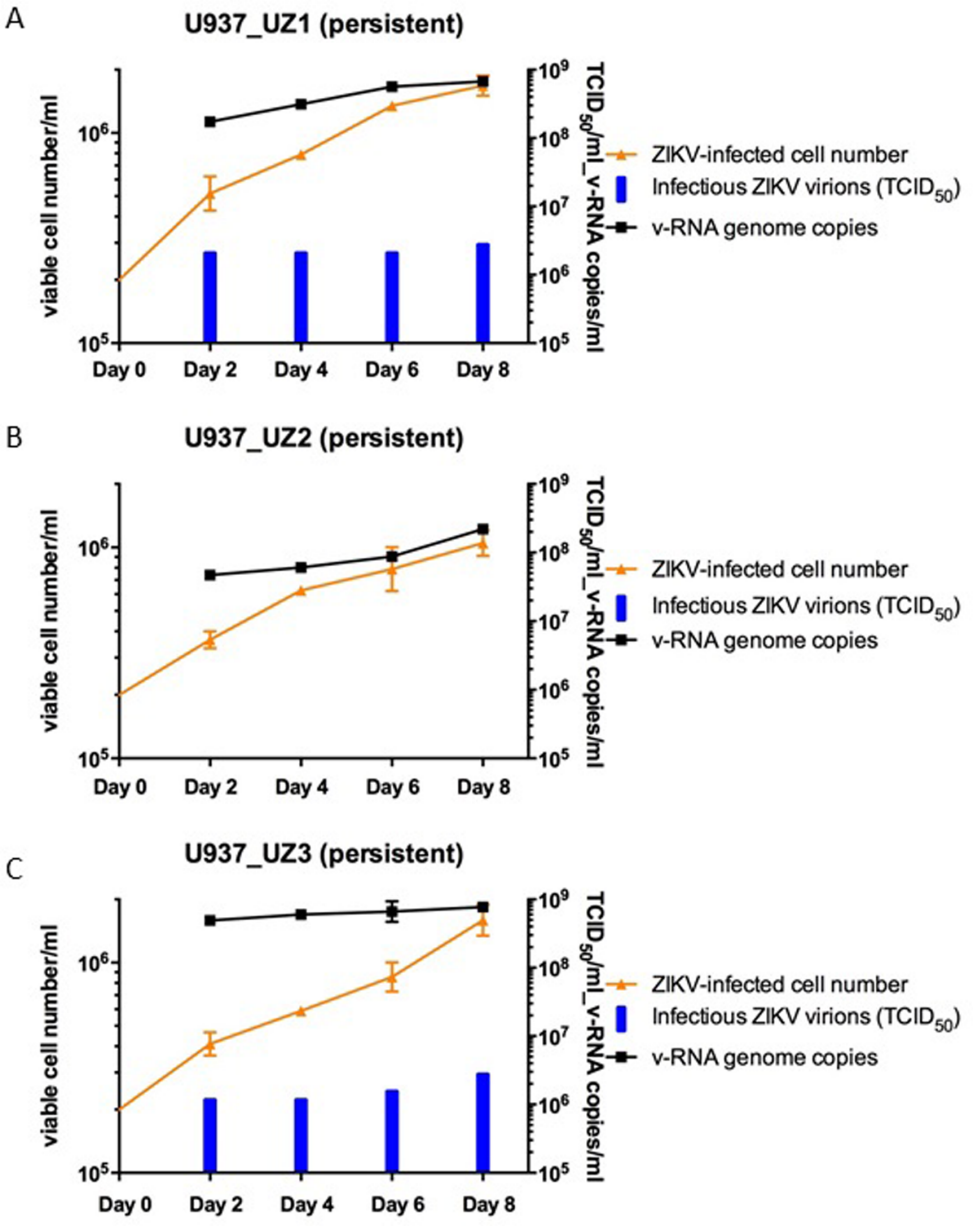
Cell growth, productions of ZIKV RNA genomes and infectious virions in cultures of the 3 continuous U937 cell lines established by further passing more than 2 months of the viable cells recovered from U937 cell cultures infected by the 3 persistent ZIKVs UZ1, UZ2 and UZ3. Cells in the persistently ZIKV-infected cultures (A) U937_UZ1 (persistent), (B) U937_UZ2 (persistent) and (C) U937_UZ3 (persistent) were harvested by centrifugation and re-suspended in fresh culture media every 2 days. The amounts of ZIKV RNA genomes and infectious virions produced into supernatants of the cultures in 2 days were quantified by qPCR and titrated by TCID_50_ assay against Vero cells.

Fig 7 (panel graphs A and C) shows that ~10^9^ copies/ml of ZIKV v-RNA genomes and ~3×10^6^ ID_50_ units/ml of infectious ZIKV virions were produced by 0.5–1×10^6^ cells/ml of cultured U937_UZ1 and U937_UZ3 cell lines every 2 days. In comparison, 1–2×10^8^ copies/ml of ZIKV v-RNA genome were also produced by 0.5–0.8×10^6^ cells/ml of cultured U937_UZ2 cell line every 2 days. However, no infectious ZIKV virions that could produce typical CPE with cytolytic necrosis in the TCID_50_ study conducted on seeded Vero cells (Panel graph B in Fig 7). The cell specificity for infectivity and pathogenicity of the 2^nd^ generation persistent ZIKVs, UZ1.2, UZ2.2 and UZ3.2, produced by the cultures of U937_UZ1, U937_UZ2 and U937_UZ3 cell lines was the same as those of the respective 1^st^ generation persistent ZIKVs, UZ1, UZ2 and UZ3. Specifically, like the 1^st^ generation persistent ZIKV UZ2, the 2^nd^ generation persistent ZIKV UZ2.2 also showed no typical CPE with cytolytic necrosis on Vero cells in TCID_50_ assay (Fig 7, panel graph B) and no infectivity against the 5 human B cell lines referred to in Table 2. However, like UZ2, UZ2.2 infected K562 cells and U937 cells effectively. UZ2.2 produced prominent CPE changes with extensive cell necrosis in the infected cultures (data not shown).

### Comparative genomics of the inoculum prototype strain MR 766 ZIKV and ZIKVs produced by acutely MR 766 ZIKV-infected U937 cells with the reported MR 766 reference genome sequence

RNA was recovered from **1)** the prototype ZIKV MR 766 viral stock provided by BEI Resources, **2)** the supernatants of the Vero cells cultures used to propagate BEI-derived MR 766 viral stocks (MR 766/Vero) to be used as the inoculums for the present study and **3)** the supernatants of the U937 cell cultures infected by MR 766 ZIKV or the persistent ZIKVs UZ1 and UZ2 for the first 1–2 weeks (early phase). Furthermore, RNA was similarly recovered from **1)** the supernatants of the 3 cultures of persistently ZIKV-infected U937 cells (U937_1-ZIKV, U937_2-ZIKV and U937_3-ZIKV) established from the cultures infected by ZIKV Strain MR 766 and **2)** the supernatants of the 3 cultures of persistently ZIKV-infected U937 cells (U937_UZ1, U937_UZ2 and U937_UZ3) established from cultures infected by the persistent ZIKVs UZ1, UZ2 and UZ3, respectively (Fig 1). The RNAs were processed for v-RNA genomic sequencing using the MiSeq platform. The variations or mutations of nucleotide sequences different from the reference ZIKV strain MR 766 genome sequence [22] were identified. Nucleotide variations or mutations with less than 20% frequency were not included for the alignment and analysis in the study. Tables 3 and 4 summarize the ZIKV genomic sequencing results showing those variations or mutations with associated amino acid changes.

**Table 3:** The summary of the ZIKV RNA genomic variants identified in the inoculum prototype MR 766 ZIKV and ZIKVs produced by the acutely and persistently-infected U937 cells on the structure protein genes in comparison with the reported MR766 reference genomic sequence [22].

	Virus ID	Culture Supernatant	Position	115*	138*	338	641	767	780	841	879	1061	1116	1132	1139	1313	1368	1387	1395	1428	1451	1460	1466–1467	1492	1619	1646	1652	1788	1790	1812	1904	1913	1913	2120	2165	2316	2328
			Type	Del	Ins	SNV	SNV	SNV	SNV	SNV	SNV	SNV	SNV	SNV	SNV	SNV	SNV	SNV	SNV	SNV	SNV	SNV	SNV	SNV	SNV	SNV	SNV	SNV	SNV	SNV	SNV	SNV	SNV	SNV	SNV	SNV	SNV
			Reference	C	-	A	G	T	G	C	C	G	C	C	G	T	T	T	T	T	G	A	GA	G	C	G	G	C	G	G	T	G	G	T	C	C	C
			Variant	-	T	G	A	C	T	T	T	A	T	A	A	A	A	C	G	A	A	G	TC	A	T	T	T	T	A	A	C	C	T	C	T	T	T
**1**	**MR 766 stock from BEI**	**MR 766 ZIKV/Vero-BEI**	**Variation associated with amino acid changes**	**Glu6fs, E6K, E7K, I8K, R9S, R10G, I11G**	**Arg12fs, R12F**		D179N, 0.130			**‒, 0.217**								**‒**, 0.550			**D449N**			**‒**, 0.067						**R596K**					**H687Y, 0.253**		S741L, 0.447
**2**	**Inoculum Prototype MR 766 ZIKV**	**MR 766 ZIKV/Vero cells culture**					D179N, 0.238			**‒, 0.264**								**‒**, 0.528						**‒**, 0.159											**H687Y, 0.268**		S741L, 0.282
**3**	**MR 766-U937-day 4**	**U937-MR 766_1 -4d culture**					D179N, 0.185			**‒, 0.305**								**‒**, 0.494						**‒**, 0.132											**H687Y, 0.314**		S741L, 0.323
**4**	**MR 766-U937-day 14**	**U937-MR 766_1 -14d culture**					D179N, 0.727			**‒, 0.200**								**‒**, 0.790						**‒**, 0.400											**H687Y, 0.350**		
**5**	**UZ1-3m**	**U937_1-ZIKV - 3m culture**								− , **1.000**			T337M, 0.966						M430R, 0.971													V603L, 0.953			**H687Y**		
**6**	**UZ1-4m**	**U937_1-ZIKV - 4m culture**											T337M, 0.844						M430R, 0.884													V603L, 0.878					
**7**	**UZ1-5m**	**U937_1-ZIKV - 5m culture**											T337M, 0.467						M430R, 0.467													V603L, 0.557					
**8**	**UZ1- U937-day 13**	**U937 infected with UZ1 (13d)**											T337M, 1.000						M430R, 1.000													V603L, 1.000					
**9**	**UZ1.2-2m**	**U937_UZ1 (persistent culture)-2m**				M78V, 0.484							T337M, 0.544				Q412R, 0.362		M430R, 0.494													V603L, 0.352	V603F, 0.625			A737V, 0.423	
**10**	**UZ1.2 -3m**	**U937_UZ1 (persistent culture)-3m**				M78V, 0.334							T337M, 0.663				Q412R, 0.339		M430R, 0.073													V603L, 0.063	V603F, 0.935			A737V, 0.311	
**11**	**UZ2-4m**	**U937_2-ZIKV - 4m culture**													E345K, 1.000	L403M, 0.854							E454 S, 0.838		H505Y, 0.842	G541W, 0.850		A561V, 0.770			F600L, 0.835						
**12**	**UZ2- U937-day 13**	**U937 infected with UZ2 (13d)**													E345K, 0.882	L403M, 0.732							E454 S, 0.615		H505Y, 1.000	G541W, 0.989		A561V, 0.971			F600L, 1.000						
**13**	**UZ2.2-2m**	**U937_UZ2 (persistent culture)-2m**						S221P, 0.429			A258V, 0.450			N342K, 0.512	E345K, 1.000										H505Y, 0.486	G541W, 0.421		A561V, 0.371			F600L, 0.546						
**14**	**UZ2.2-3m**	**U937_UZ2 (persistent culture)-3m**						S221P, 0.609			A258V, 0.611			N342K, 0.472	E345K, 1.000										H505Y, 0.435	G541W, 0.422		A561V, 0.476			F600L, 0.433						
**15**	**UZ3-5.5m**	**U937_3-ZIKV—5.5m culture**							R225M, 0.222			G319S, 0.333								M441K, 0.556		K452E, 0.571					D516Y, 0.429		E562K, 0.517					T672H, 0.286			
**16**	**UZ3 - U937-day 15**	**U937 infected with UZ3 (15d)**							R225M, 0.674			G319S, 0.348								M441K, 0.619		K452E, 0.366					D516Y, 0.275		E562K, 0.710					T672H, 0.327			
**17**	**UZ3.2–2.5m**	**U937_UZ3 (persistent culture)-2.5m**							R225M, 1.000											M441K, 1.000									E562K, 1.000								
**18**	**UZ3.2–4.5m**	**U937_UZ3 (persistent culture)-4.5m**							R225M, 1.000											M441K, 1.000									E562K, 1.000								
**Encoded Proteins**			**Mature peptide**	**Anchored capsid protein C**			**Membrane glycoprotein precursor M**			**Membrane glycoprotein M**		**Envelope protein E**																									
			**nt sequence range**	**107–418; 107–472**			**473–751; 473–976**			**752–976**		**977–2476**																									
			**AA range**	**1–104; 1–122**			**123–215; 123–290**			**215–290**		**291–790**																									

**Note:** The coordination of the variants is corresponding to the reference genome sequence of the ZIKV MR 766 Uganda prototype retrieved from GenBank (Accession number NC_012532).

SNV stands for single nucleotide variation.

*: Single nucleotide insertion (Ins)/ deletion (Del) caused frameshift (fs). Light gray shadings: variants found in the inoculum prototype MR 766 ZIKV genome, which were different from the reference genome NC_012532.

The variations from the same set of experiment have identical background color. Empty cells indicate the absence of the variants on the corresponding positions; the number along with the amino acid change is its variation ratio found in the sample, the amino acid changes with variation ratio >0.99 were not labeled.

The synonymous nucleotide variant is indicated using a dash mark followed by its variation ratio. The three rows on the bottom indicate the spans of the encoded proteins on the viral polypeptide and which’s corresponding positions of the viral RNA genome.

**Table 4:** The summary of the ZIKV RNA genomic variants identified in the inoculum prototype MR 766 ZIKV and ZIKVs produced by the acutely and persistently-infected U937 cells on the non-structure protein genes in comparison with the reported MR766 reference genomic sequence [22].

	Virus ID	Culture Supernatant	Position	2841	2511	2739	3650	3869	4257	4325	4822	5302	5444	6297	6507	6549	6569	6612	6659	6868	7157*	7160	7166*	7370	7519	7526	7563	7782	7815	7838	7851	7874	8030	8289	8845	8856	10126	10331	10698
			Type	SNV	SNV	SNV	SNV	SNV	SNV	SNV	SNV	SNV	SNV	SNV	SNV	SNV	SNV	SNV	SNV	SNV	Ins	SNV	Del	SNV	SNV	SNV	SNV	SNV	SNV	SNV	SNV	SNV	SNV	SNV	SNV	SNV	SNV	SNV	Ins
			Reference	A	A	T	G	C	C	A	G	G	A	C	A	G	C	C	T	G	-	A	G	A	G	G	C	C	C	A	A	T	C	G	A	T	C	T	-
			Variant	G	G	C	T	T	T	G	A	T	T	T	G	A	T	T	C	A	T	G	-	G	A	A	T	T	T	C	T	C	T	C	G	C	T	C	C
**1**	**MR 766 stock from BEI**	**MR 766 ZIKV/Vero-BEI**	**Variation associated with amino acid changes**					L1255F, 0.089			**‒**, 0.090		**N1780Y**								**Met2353fs, M2352Y, H2353A,**		**Try2354fs, G2354W**		**‒**, 0.558					**I2578L**	**E2582V**			**C2728S**	**‒**, 0.144		**‒**, 0.568	**‒**	**‒**
**2**	**Inoculum Prototype MR 766 ZIKV**	**MR 766 ZIKV/Vero cells culture**						L1255F, 0.100			**‒**, 0.164														**‒**, 0.550										**‒**, 0.268		**‒**, 0.568		
**3**	**MR 766-U937-day 4**	**U937-MR 766_1 -4d culture**						L1255F, 0.101			**‒**, 0.139														**‒**, 0.741										**‒**, 0.229		**‒**, 0.548		
**4**	**MR 766-U937-day 14**	**U937-MR 766_1 -14d culture**									**‒**, 0.400														**‒**, 0.833										**‒**, 0.364		**‒**, 0.563		
**5**	**UZ1-3m**	**U937_1-ZIKV - 3m culture**													E2134G, 0.580																								
**6**	**UZ1-4m**	**U937_1-ZIKV - 4m culture**													E2134G, 0.720																								
**7**	**UZ1-5m**	**U937_1-ZIKV - 5m culture**													E2134G, 0.692																								
**8**	**UZ1- U937-day 13**	**U937 infected with UZ1 (13d)**													E2134G,1.000																								
**9**	**UZ1.2-2m**	**U937_UZ1 (persistent culture)-2m**								I1407V, 0.187					E2134G,0.897													A2559V, 0.207											
**10**	**UZ1.2 -3m**	**U937_UZ1 (persistent culture)-3m**								I1407V, 0.591					E2134G, 0.621			T2169I, 0.316										A2559V, 0.600											
**11**	**UZ2-4m**	**U937_2-ZIKV - 4m culture**							A1384V, 0.844					T2064I, 0.825		R2148Q, 0.800			F2185L, 0.580			Y2352C, 0.819		I2422V, 0.818															
**12**	**UZ2-U937-day 13**	**U937 infected with UZ2 (13d)**														R2148Q, 0.909			F2185L, 0.941			Y2352C, 0.745		I2422V, 0.767															
**13**	**UZ2.2-2m**	**U937_UZ2 (persistent culture)-2m**													E2134G, 0.655		P2155S, 0.650		F2185L, 0.471										A2570V, 0.438			Y2590H, 0.478							
**14**	**UZ2.2-3m**	**U937_UZ2 (persistent culture)-3m**			E802G, 0.594	V878A, 0.690									E2134G, 0.467		P2155S, 0.480		F2185L, 0.500								T2486I, 0.333		A2570V, 0.575			Y2590H, 0.564	L2642F, 0.450			I2917T, 0.560			
**15**	**UZ3-5.5m**	**U937_3-ZIKV—5.5m culture**										E1732D, 0.400			E2134G, 0.858					M2254I, 0.333						G2474S, 0.400													
**16**	**UZ3 - U937-day 15**	**U937 infected with UZ3 (15d)**													E2134G, 0.753					M2254I, 0.753																			
**17**	**UZ3.2–2.5m**	**U937_UZ3 (persistent culture)-2.5m**		Y912C, 0.364			V1182L, 0.714								E2134G, 1.000					M2254I, 1.000																			
**18**	**UZ3.2–4.5m**	**U937_UZ3 (persistent culture)-4.5m**		Y912C, 0.750			V1182L, 0.286								E2134G, 1.000					M2254I, 1.000																			
**Encoded Proteins**			**Mature peptide**	**NS1**			**NS2A**		**NS2B**		**NS3**				**NS4A**					**Protein 2K**	**NS4B**							**NS5**											**3'UTR**
			**nt sequence range**	**2477–3532**			**3533–4210**		**4211–4600**		**4601–6451**				**6452–6832**					**6833–6901**	**6902–7654**							**7655–10363**											**10367–10794**
			**AA range**	**791–1142**			**1143–1368**		**1369–1498**		**1499–2115**				**2116–2242**					**2243–2265**	**2266–2516**							**2517–3419**											

**Note:** The coordination of the variants is corresponding to the reference genome sequence of the ZIKV MR 766 Uganda prototype retrieved from GenBank (Accession number NC_012532).

SNV stands for single nucleotide variation.

*: Single nucleotide insertion (Ins)/ deletion (Del) caused frameshift (fs). Light gray shadings: variants found in the inoculum prototype MR 766 ZIKV genome, which were different from the reference genome NC_012532.

The variations from the same set of experiment have identical background color. Empty cells indicate the absence of the variants on the corresponding positions; the number along with the amino acid change is its variation ratio found in the sample, the amino acid changes with variation ratio >0.99 were not labeled.

The synonymous nucleotide variant is indicated using a dash mark followed by its variation ratio. The three rows on the bottom indicate the spans of the encoded proteins on the viral polypeptide and which’s corresponding positions of the viral RNA genome.

The previously reported prototype strain MR 766 ZIKV reference genomic sequence [22] with its nucleotide positions was used as the coordination in our comparative ZIKV genomic study. In comparison with the reference ZIKV genome sequence, the BEI-derived prototype strain MR 766 ZIKV and the inoculum MR 766 ZIKV strain propagated in Vero cells (MR 766/Vero) in our study had a deletion at position 115 and an insertion at position 138. These two close frameshifts resulted in a total 7 amino acid changes in this specific region of capsid protein C. In addition, the BEI-derived inoculum MR 766/Vero strain had 2 nucleotide mutations that occurred at positions 1451 and 1812 in the envelop protein E gene, and one mutation occurred at position 5444 in the NS3 gene. Moreover, a single nucleotide insertion and deletion caused frameshifts at positions 7157 and 7166 of the NS4B gene, and 3 mutations occurred at positions 7838, 7851 and 8289 in the NS5 gene. All 10 variations or mutations identified in the respective genes would result in amino acid changes. There was also one nucleotide insertion at position 10698 in the 3’ end untranslated region (3’UTR). The variations identified at all these positions in the BEI-derived ZIKV prototype strain MR 766 were highly stable. They were consistently conserved in all the genomes of ZIKVs propagated in the cultures of Vero cells or produced in the cultures of U937 cells following both acute and persistent ZIKV infections (Tables 3 and 4). Remarkably, very few sequence heterogeneities were found at these positions among the raw reads generated using genome sequencing of all the ZIKV samples examined in this study.

### Comparing genome sequences of ZIKVs produced by U937 cells after establishing the 1st and the 2nd rounds of persistent ZIKV infection with that of the inoculum prototype MR 766 ZIKV strain

Genomic sequencing studies of the BEI prototype ZIKV MR 766 viral stock and the inoculum MR 766/Vero revealed significant percentages of sequence reads with heterogeneity at positions 641, 841, 1387, 1492, 2165, 2328, 3869, 4822, 7519, 8845, 10126 (Tables 3 and 4, samples #1 and #2). Sequence heterogeneity at these positions continued to be present in the genomes of ZIKVs produced in the U937 cells cultures infected by the infectious viral stock inoculum MR 766/Vero in the early phase (1–2 weeks, samples # 3 and 4). However, heterogeneity of sequence reads disappeared completely at all these positions in the genomes of all 3 persistent ZIKVs (UZ1, UZ2 and UZ3, samples # 5–7, 11 and 15) produced by the U937 cells after establishment of ZIKV persistence in culture. The sequence heterogeneity continued to be absent at all these positions in the genomes of the 2^nd^ generation persistent ZIKVs (UZ1.2, UZ2.2 and UZ3.2, samples # 9, 10, 13, 14, 17 and 18), despite the appearance of new sequence variations and heterogeneity at different gene positions (Tables 3 and 4).

Genomic analysis revealed that few new variations or mutations would be seen in the genomes of ZIKVs produced in U937 cell cultures following infections with either the inoculum prototype strain MR 766 ZIKV or the 3 persistent ZIKVs in the first 1–2 weeks (early phase, samples # 3, 4, 8, 12 and 16). However, in comparison with the genome sequence of the inoculum prototype strain MR 766 ZIKV, genomic analysis revealed 3 clearly different sets (color coded in Tables 3 and 4) of new variations or mutations with associated amino acid changes in the respective genomes of persistent ZIKVs, UZ1 (green), UZ2 (blue) and UZ3 (red) produced by the 3 continuous U937 cell lines after they established ZIKV persistence. These prominent new mutations occurred at distinct positions in the membrane glycoprotein M gene, envelope protein E gene and different NS genes of the 3 genomes of persistent ZIKVs, UZ1, UZ2 and UZ3 (Tables 3 and 4). Specifically, a set of mutations at positions 1116, 1395 and 1913 of envelop E gene as well as 6507 of NS4A gene was found in the genome of persistent ZIKV UZ1. A separate set of mutations at positions 1139, 1619, 1646, 1788 and 1904 of envelope E gene, as well as 6659 of NS4A gene was found in the genome of persistent ZIKV UZ2. A third set of characteristic mutations at positions 1428 and 1790 of envelope E gene as well as 6507 of NS4A gene were found in the genome of persistent ZIKV UZ3. In addition, UZ3 had mutations at positions 780 of membrane glycoprotein M gene and 6868 of protein 2K gene. These mutations developed in genomes of the established persistent ZIKVs appeared to be rather stable. Our study showed that they persisted in the genomes of ZIKVs (UZ1-3m, UZ1-4m and UZ1-5m) produced by persistently ZIKV-infected culture U937_1 ZIKV passed weekly in cultures for 3 to 5 months (Table 3, samples 5–7).

Comparative ZIKV genomics showed that many of these mutations found in the 3 respective genomes of the 1^st^ generation persistent ZIKVs continued to be present in the respective genomes of the 2^nd^ generation persistent ZIKVs (UZ1.2, UZ2.2 and UZ3.2) produced by U937 cells that established a new round of ZIKV persistence following infections of the 1^st^ generation of persistent ZIKVs UZ1, UZ2 and UZ3 in culture (Tables 3 and 4). However, some of the mutations disappeared and new mutations appeared. Amino acid changes not found in the genomes of the 1^st^ generation of persistent ZIKVs UZ1, UZ2 and UZ3 were seen at some positions of Envelop E gene and NS genes in the genomes of the 2^nd^ generation of persistent ZIKVs UZ1.2, UZ2.2 and UZ3.2. More nucleotide variations or mutations with associated amino acid changes could be seen in the genomes of persistent ZIKVs produced by the persistently ZIKV-infected culture U937_UZ1, U937_UZ2 passed weekly in cultures for a longer period (Tables 3 and 4).

## Discussion

We examined human blood cell lines with different differentiation properties for possibly supporting continued propagation of ZIKV and developing ZIKV persistence. Consistent with the finding of a previous study [23], we found that the prototype ZIKV MR 766 strain infected human histiocytic lymphoma-originated U937 cells with very low efficiency and produced no detectable CPE changes with cytolytic necrosis in the ZIKV-infected cultures (Fig 2). However, our study revealed that supporting continuous growth of viable U937 cells in the cultures with low grade ZIKV infection would consistently lead to persistently ZIKV-infected U937 cell lines expressing ZIKV-specific antigens (Fig 3) and constantly producing both ZIKV RNA genomes and infectious virions (Fig 5). An additional study involving human cells of different developmental characteristics to infection using different ZIKV strains will be reported separately. Our preliminary results were in general consistent with those of other reports that an African ZIKV isolate (MR 766 strain) is more infectious and cytopathogenic than other isolates circulating in the Americas [24, 25] in the context of experimental infections involving various human host cells.

There were dynamic interplays between the ZIKVs and the infected human host cells in the intricate process of establishing viral persistence. However, the nature of viral population selection and host cell adaptation might be very different between the 2 respective rounds of establishing ZIKV persistence in U937 cells described in our study. Since the inoculum prototype strain MR 766 ZIKV could only infect and propagate in U937 cells at a very low rate, the slow kinetics of ZIKV infection may have resulted in the activation of various antiviral mechanisms in the U937 cells. This is consistent with the finding that none of the U937 cells in the three MR 766-infected cultures (Fig 2) had CPE changes and underwent cytolytic necrosis. In the prolonged process of establishing the 1^st^ round of viral persistence in U937 cells, the specific groups or clones of ZIKVs with genetic variations or mutations in the key genes that enabled the viruses to effectively infect and propagate in the U937 cells, which had likely activated cellular antiviral mechanisms, were being selectively enriched from the heterogeneous population of the inoculum prototype strain MR 766 ZIKV. Interestingly, the 3 independently established persistent ZIKVs were composed of 3 highly selected “groups” or “clones” of ZIKV carrying 3 distinct sets of mutations that were not found in the genome of the inoculum prototype strain MR 766 ZIKV. The pressure for selecting the ZIKV clones carrying a special set or combination of mutations would likely be needed to maintain the viral persistence in these U937 cell lines. The mutations that occurred were stably maintained in the ZIKVs produced by the persistently ZIKV-infected U937 cell lines passed weekly in culture for months (Tables 3 and 4), showing that the genetic changes introduced are essential for survival of the virus.

In the process of establishing the 2^nd^ round of persistently ZIKV-infected U937 cell lines, fresh U937 cells were infected with the 3-established persistent ZIKVs UZ1, UZ2 and UZ3 that propagated effectively in U937 cells that had their antiviral mechanisms activated. Thus, large numbers of naïve U937 cells in the cultures infected by the 3 persistent ZIKVs quickly became IFA-positive for ZIKV antigen, developed prominent CPE changes and underwent cytolytic necrosis (Fig 5). The selection pressure in establishing the 2^nd^ round of ZIKV persistence in cultures of U937 cells was mainly on selecting the special members or subpopulations of U937 cells that could sustain and adapt to growth in the cultures with active infections of ZIKVs. The process was no longer selecting the unique clones of ZIKVs with the specific biological properties. Thus, the 2^nd^ generation persistent ZIKVs, UZ1.2, UZ2.2 and UZ3.2 produced by the 3 continuous U937 cell lines that established viral persistence following infections of the 1^st^ generation of persistent ZIKVs, UZ1, UZ2 and UZ3 (Fig 1) retained many of the genetic mutations of the respective persistent ZIKVs (Tables 3 and 4). Not surprisingly, they also shared very similar properties in cell infectivity or pathogenicity of the respective 1^st^ generation of persistent ZIKVs. However, both losing and gaining mutations at new genomic sites could also be seen in the genomes of the 2^nd^ generation persistent ZIKVs UZ1.2, UZ2.2 and UZ3.2, especially when the 3 persistently ZIKV-infected U937 cell lines U937_UZ1, U937_UZ2 and U937_UZ3 were being passed in culture for longer periods of time (Tables 3 and 4).

The nucleotide variations or mutations identified in the genomes of the persistent ZIKVs UZ1, UZ2 and UZ3 resulted in specific amino acid changes at different positions of the key viral envelope protein E gene and NS4A genes (Tables 3 and 4). UZ2 had mutations at positions 7160 and 7370 of NS4B gene. UZ3 also had a mutation at position 780 of the membrane glycoprotein M gene and a mutation at position 6868 of the protein 2K gene between NS4A and NS4B genes. Each mutation or sets of mutations could be playing a vital role in the establishment and maintenance of ZIKV persistence in U937 cells. In this context, it is important to note that a single nucleotide variation at position 2165 of the envelop protein E gene, presenting with only low frequency in the inoculum MR 766 ZIKV genome, was shared without any sequence heterogeneity by all the genomes of persistent ZIKVs produced by U937 cell lines that emerged in both rounds of ZIKV persistence (Tablea 3 and 4). The mutation at this position could be highly critical in both developing and maintaining virus persistence in the ZIKV-infected human U937 cell lines. Both restricted expression and mutations of Flavivirus envelop E protein were reported previously to play a significant role in developing persistence of various Flaviviruses [26–29]. Mutations in the NS genes were also thought to be associated with viral persistence in WNV [30]. Other than playing a significant role in establishment and/or maintenance of viral persistence in the infected host cells, each of these identified mutations, individually or in various combinations could also play a prominent role in affecting the persistent ZIKVs’ specificity in cell infectivity and pathogenicity. The mutations occurred in these genes were reported to be associated with cell infectivity and/or pathogenicity of ZIKV and WNV [31–36]. The biological significance of these mutations identified in the genomes of persistent ZIKVs warrants further studies.

More than 80 percent of natural ZIKV infections are asymptomatic low-grade infections and ZIKV can persist in the infected individuals for a prolonged period. Our *in-vitro* study of ZIKV persistence may have important clinical implications and further our understanding of ZIKV evolution. A recent report [37] indicating that monocytes appeared to be the main targets of Zika virus infection in human PBMCs. Studying molecular mechanisms by which ZIKV establishes persistence in U937 cells of human monocytic/histiocytic origin should have high physiological and clinical relevance. The study showed that both viral and cellular mechanism played a role in the development of ZIKV persistence in U937 cells. One process involved selection of unique ZIKV clones with mutations in key viral genes enabling the viruses to continuously infect and proliferate in the human blood cells in which anti-viral mechanisms were activated. The other process involved selection of special human blood cells with adaptive metabolic functions enabling the cells to continuously grow without undergoing apoptosis or cytolysis, despite active propagation of ZIKVs. The persistent ZIKVs produced by the human U937 cells after independently developing viral persistence each time had properties not only distinct from the original ZIKV, but also among themselves in the specificity of cell infection and pathogenicity. The establishment of persistently ZIKV-infected human cell lines in our study may have the following implications. **(1)** They may facilitate future studies to determine molecular and biological bases of viral persistence in human cells. The gene expression profiles of human cells with persistent ZIKV infections can be analyzed in detail to explore the critical pathways that might accommodate or confer viral persistence. **(2)** The “permanent” human cell lines continuously producing ZIKV in culture may serve as a useful tool or platform for rapid screening of drugs or biologics as potential anti-viral therapeutics. **(3)** They may provide a useful model to evaluate various pathogen reduction methods or protocols developed to improve blood and tissue safety. **(4)** The persistently ZIKV-producing human cell lines may also be useful for vaccine development against ZIKV outbreaks.

## Materials and methods

### Virus and cells

Zika virus (ZIKV), MR 766 strain (NR-50065) and Genomic RNA from Zika virus, MR 766 (NR-50085) were obtained from BEI Resources, NIAID/NIH. Human cell lines CCRF-SB (B lymphoblast, ATCC CCL-120), CCRF-CEM (T lymphoblast, ATCC CRM-CCL-119), CCRF-HSB2 (T lymphoblast, ATCC CCL-120.1), K562 (lymphoblast, ATCC CCL243), U937 (histiocytic lymphoma, ATCC CRL-1593.2), THP-1 (monocyte, ATCC TIB202), HL-60 (promyeloblast, ATCC CCL240) and KG-1 (lymphoblast, ATCC CRL-8031) as well as Vero cells (African green monkey kidney cells, ATCC CCL-81) were obtained from ATCC. RPMI-8392 (B cell line 8392, GM03638) and RPMI-8402 (T cell line 8402, GM03639) were obtained from NIGMS Human Genetic Mutant Cell Repository. Molt-3 (human T lymphoblast) was obtained from Biotech Research Labs, Rockville MD. The human B cell line K2267-Mi was previously established and characterized in our laboratory [38]. EBV-positive human lymphoblastoid B-cell lines 824–00 was developed from spontaneous immortalization of healthy blood donor PBMC and 4695EB was established from human B cells immortalized by EBV strain B95-8 transformation in our laboratory. Unless specified otherwise, cell cultures were grown in RPMI 1640 medium supplemented with 10% fetal bovine serum (FBS) and 1% penicillin-streptomycin solution at 37°C and 5% CO_2_.

### Propagation of the ZIKV MR 766 working stock

A T75 culture flask with 90% confluent Vero cells kept in 10 ml of RPMI 1640 medium containing 2% FBS (R2 medium) was infected with ZIKV MR 766 obtained from BEI Resources at multiplicity of infection (MOI) of 0.1–0.01 according to the package description. The ZIKV-infected Vero cell culture was incubated in a CO2 incubator at 37°C and monitored daily for the appearance of viral CPE. The culture supernatant was harvested by centrifugation, when ~80% of cells detached from the culture surface of the flask after developing CPE. The culture supernatant was filtered to remove cell debris, aliquoted, tittered and stored at -80°C as the ZIKV working stock to be used in the subsequent experiments.

### Infection of human hematopoietic cell lines with Zika virus

About 2×10^6^ cells were harvested from an actively growing suspension cell culture by centrifugation in a 15-ml tube. Cells were washed once with R2 medium and collected by centrifugation. The washed cells were suspended in 0.3 ml of R2 medium containing 4×1060020030p.f.u. of ZIKV MR 766, and incubated at 37°C for 2 hours with frequent shaking to adjust the virus inoculum to ~2 MOI (if not otherwise specified). After incubation, cells were washed with fresh R2 medium for 3 times, and cultured in 10 ml of RPMI 1640 medium supplemented with 10% FBS in a T-25 culture flask kept at 37°C in 5% CO_2_ incubator. The starting cell concentration in culture was normally adjusted to 1–2×10^5^ cells/ml. At the designated time points, 0.5 ml of each suspension cell culture was sampled to perform viable cell count and IFA study to identify and quantify Flavivirus group antigen-positive cells. Culture supernatants were stored at -80°C for quantitative measurements of infectious ZIKV virions by endpoint dilution assay as well as v-RNA genome copies by real-time reverse transcription-PCR.

### Endpoint dilution assay (TCID_50_)

Infectious titer was measured as the 50% tissue culture infective dose (TCID_50_), using Vero cells. Exponentially growing Vero cells were seeded in a 96-well culture plate at 2×10^4^ cells/well in RPMI 1640 medium containing 10% FBS one day before performing the assay. Aliquots of supernatants collected previously from ZIKV-infected cell cultures at the designated time points were 10-fold serially diluted in R2 medium. In the assay, the culture medium of the prepared 96-well plates of Vero cells was aspirated and 20 μl of serially diluted culture supernatant of ZIKV working stock was added into each well. Eight wells were inoculated for each dilution of the culture supernatant tested. After incubating the plates at 37°C for 2 hours for viral absorption, 150 μl fresh R2 medium were added into each well. The plates were incubated in a CO2 incubator at 37°C and followed for 4 to 6 days. At the end of assay, the numbers of culture wells showing clear viral CPE was recorded. The TCID_50_ titer was calculated based on the method described by Hierholzer & Kilington in the first edition of the Virology Methods Manual.

### Immunofluorescence assay

To prepare cell samples for immunofluorescence assay (IFA), 0.5 ml of the cell suspension was harvested from a cell culture and centrifuged at 1000 rpm for 10 min in a microfuge tube. The cell pellet was washed with 1ml of phosphate buffered saline (PBS). After centrifugation, the cell pellet was re-suspended in 50 μl of PBS and 10–20 μl of cell suspension was dotted on a microscopic slide. The cell slides were air-dried and then fixed with mixture of methanol: acetone (1:1) for 5 min at room temperature. To detect the presence of ZIKV antigenic proteins in cells, Anti-Flavivirus Group antigen monoclonal antibody (Clone D1-4G2-4-15, BEI Resources) was used as the primary antibody of IFA to detect cells that were positive for producing ZIKV antigen. Cell dots were blocked for 30 min with a blocking solution (KPL, Gaithersburg MD, USA), and 1% bovine serum albumin (BSA) in PBS containing 100 μg/ml of human immunoglobulin. Cell smears on the slides were then incubated with primary antibody (1:300 dilutions in 1% BSA-PBS) at room temperature for 30min in a humidified chamber. After washing with PBS, 3 times, the cell smears were incubated with a secondary antibody, Alexa Fluor 488 conjugated goat-anti-mouse IgG (1:400) (Jackson ImmunoResearch Labs, PA) for 30min. The slide was washed with PBS, 3 times and then once with distilled water. The slides were air dried and mounted with glycerol-PBS mixture containing DAPI (4’,6-diamidino-2-phenylindole, Sigma, Mo). The cell images were captured using an up-right immunofluorescent microscope with attached digital camera system (Olympus BX51).

### Electron microscopy

For transmission electron microscopy (TEM), cell samples were centrifuged at 2000 rpm for 10 min in a microfuge tube, a small cell pellet was then fixed with 2.5% glutaraldehyde in 0.1M sodium cacodylate buffer pH7.4 at 4°C for overnight and post-fixed with 1% OsO_4_ in PBS. Samples were dehydrated in a series 50%, 70%, 95% of ethanol for 10 min each, 3 times in 100% ethanol for 10 min each, and 3 times in propylene oxide for 10 min each. The samples were then processed for infiltration of propylene with Epon12 overnight at RT and embedded in Epon12 epoxy resin at 60 ^o^C in the oven for 48 hours. Ultrathin sections were cut by ultra-microtome, stained with uranyl acetate and lead citrate, and then examined with a Zeiss Libra 120 Plus transmission electron microscope. The electron micrographs were taken with a Gatan US1000XP digital camera.

### Real-time one-step reverse transcription-quantitative PCR (RT-qPCR)

Quantification of v-RNA of ZIKV genome was performed by a real-time RT-PCR assay using ZIKV-specific primer sets. Total RNA of a culture supernatant was extracted from 140 μl of sample stored at -80°C using QIAamp Viral RNA Mini Kit (Qiagen, Dusseldorf Germany), following the manufacturer’s protocol. The v-RNA copy numbers in purified RNA were quantified using iTaq Universal SYBR Green One-Step Kit (Bio-Rad, CA USA) and Bio-Rad CFX96 system (Bio-Rad, CA USA). Each reaction mixture of one-step RT-qPCR contained 5 μl of purified RNA, 10 μl of iTaq universal SYBR Green reaction mix, 0.25 μl of iScript reverse transcriptase, 0.5 μl each of 10uM forward and reverse primer, and 4.25 μl of nuclease-free water, to a final volume of 20 μl. The RT-PCR was started from 50°C reverse transcription step for 30 min and followed by PCR amplification with 95°C pre-heat for 1 min and then 45 two-step thermocycles at 95°C for 10 sec and 57°C for 30 sec. The ZIKV-specific primer pair used in the RT-qPCR reaction was described previously [39]. The sequences of the forward and reverse primers are: 5’-CCTTGGATTCTTGAACGAGGA-3’ and 5’-AGAGCTTCATTCTCCAGATCAA-3’. The standard curve for estimating v-RNA copy number present in the tested sample was calculated by fitting the Ct value to a strand curve. The standard curve was generated by RT-PCR run against six RNA samples prepared from a10-fold serial dilution of the ZIKV MR 766 genomic RNA solution containing 1.1×10^6^ ZIKV RNA genome copies/μl provided by BEI.

### Viral RNA preparation for viral genome sequencing

RNAs of viral particles were extracted from the cell culture supernatant. Culture supernatant, 50 ml, from acute or persistent infected cells was harvested. The supernatant was centrifuged at 2000g for 10 min, dispersed and passed through a 0.22 μm filter to further remove cell residuals. The supernatant was chilled on an ice-bath and 20 ml of 20% PEG (polyethylene glycol)-8000 solution (with 2.5M NaCl) was added into culture supernatant. The PEG-supernatant mixture was stored at 4°C, overnight. The overnight chilled mixture was centrifuged at 3000g, 4°C for 30 min. The supernatant was discarded and the precipitate was re-suspended in100 μl of PBS. Trizol Reagent, 300 μl, was added into the viral suspension to extract viral RNA using Direct-zol MiniPrep kit (Zymo Research, CA, USA), following the procedures provided by the manufacturer. The extracted RNA samples were quality checked and quantified by RNA pico chips of 2100 Bioanalyzer (Agilent, CA, USA).

### RNA-Seq of viral genome

The RNAs from supernatants were used to generate libraries using Illumina TruSeq stranded total RNA sample prep kit, following the manufacturer’s standard procedures. In brief, total RNA were fragmented in the presence of divalent ions at 94°C for 8 minutes. Fragmented RNAs were reverse-transcribed into first-strand cDNAs, and then second-strand cDNAs. The double strand cDNAs were adenylated at the 3’ ends, ligated to indexed sequencing adaptors, and amplified for 15 cycles. The cDNA Libraries were loaded in one MiSeq Nano Flow Cell using MiSeq Reagent Nano kit for a 100-cycle paired-end sequencing on the MiSeq sequencer (Illumina, San Diego, CA, USA).

### Analysis of the viral genome variants

The fastq files generated from the Illumina MiSeq sequencer were used for viral genome variant analysis. All the sequence analyses were performed by the software package CLC Genomics Workbench version 9.5.3. The raw reads were trimmed to remove the regions with more than two ambiguous bases, and the regions having 5% of the bases with quality score lower than 20. The trimmed reads were mapped to the reference sequence of the ZIKV MR 766 Uganda prototype (GenBank Accession number: NC_012532) with the mapping parameter: match score = 1, Mismatch cost = 2, insertion cost = 3, deletion cost = 3, mapped length fraction>0.5, and similarity fraction>0.8. The local realignment was applied to realign the unaligned ends from the reads mapping. The aligned reads tracks from the local realignment were used for Low Frequency Variant Detection with the parameter: Ploidy = 1, required variant probability = 80%, the minimum coverage = 5, minimum reads count = 2, minimum frequency = 1%. The reads mapping BAM file of each analyzed sample listed on Tables 3 and 4 can be downloaded from Dryad repository, doi:10.5061/dryad.7r7812c.

## Supporting information

S1 Fig **Electron micrographs of persistently ZIKV-infected U937_ZIKV-1 cell line (A) and persistently ZIKV-infected U937_ZIKV-2 cell line (B) with size bars.** Insert Boxes: Electron-dense virus-like particles (red arrows) in higher magnification. Yellow arrow head: Cell membrane. N: Nucleus. M: Mitochondria.
(TIF)

S2 Fig Atypical CPE without cytolysis seen in the TCID_50_ assay wells seeded with Vero cells and inoculated (<10^1^ dilution) with low dilutions of supernatant from the U937_ZIKV-2 culture. **A1 (100X) and A2 (400X): Vero cells seeded in the TCID_50_ assay well inoculated with supernatant of the control U937 cell culture showed no CPE changes** at day 3. Vero cells in the assay wells inoculated with the inoculum prototype MR 766 strain ZIKV and supernatant of the persistently ZIKV-infected U937_ZIKV-1 cell culture showed prominent CPE changes at day 3. **B1 (100X) and B2 (400X):** Vero cells seeded in the TCID_50_ assay well inoculated with supernatant of the control U937 cell culture showed no CPE-associated cytolysis at day 5. Vero cells in the assay wells inoculated with the inoculum prototype MR 766 strain ZIKV and UZ1 from supernatant of the persistently ZIKV-infected U937_ZIKV-1 cell culture showed prominent CPE with extensive cytolysis and cell sloughing at day 5. Vero cells in the assay wells inoculated with UZ2 from supernatant of the persistently ZIKV-infected U937_ZIKV-2 cell culture had clusters of cells with atypical CPE changes at day 5. However, no cytolysis or cell sloughing was seen in the well (A1 and A2). Vero cells in these TCID_50_ assay wells had no cytolysis or cell sloughing even at day 7 post inoculation of UZ2 from supernatant of the persistently ZIKV-infected U937_ZIKV-2 cell culture (B1 and B2).
(TIF)

S3 Fig **Cell growth, numbers of cells IFA-positive for ZIKV antigen, productions of ZIKV RNA genomes and infectious virions in cultures of K562 cells infected with the 3 persistent ZIKVs UZ1(A), UZ2 (B) and UZ3 (C).** The cultures of K562 cells (2×10^5^ cells/ml) were infected with the 3 persistent ZIKVs (~10^7^ copies of ZIKV v-RNA genome/ml) prepared from the culture supernatants of persistently ZIKV-infected U937 cell lines U937_1-ZIKV, U937_2-ZIKV and U937_3-ZIKV. Prominent CPE with extensive cytolysis and cell loss were seen in all the 3 cultures infected with the 3 persistent ZIKVs UZ1, UZ2 and UZ3. The amounts of ZIKV RNA genomes and infectious virions produced into supernatants of the ZIKV-infected cultures were quantified by qPCR and titrated by TCID_50_ assay against Vero cells.
(TIF)
