# Supplementary figures and images for: Comparative genomics, infectivity and cytopathogenicity of Zika viruses produced by acutely and persistently infected human hematopoietic cell lines
Source: PLoS One. 2018 Sep 7;13(9):e0203331. doi: 10.1371/journal.pone.0203331 (PMC6128475; doi:10.1371/journal.pone.0203331)

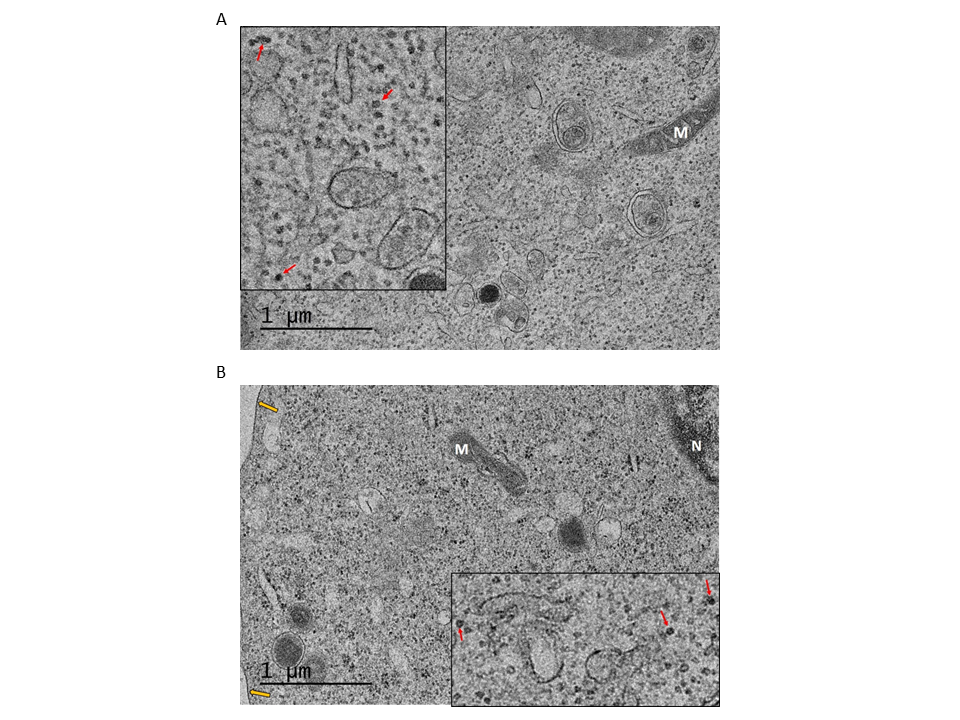

Supplement: S1 Fig — Electron micrographs of persistently ZIKV-infected U937_ZIKV-1 cell line (A) and persistently ZIKV-infected U937_ZIKV-2 cell line (B) with size bars. Insert Boxes: Electron-dense virus-like particles (red arrows) in higher magnification. Yellow arrow head: Cell membrane. N: Nucleus. M: Mitochondria. (TIF) [file pone.0203331.s001.TIF]

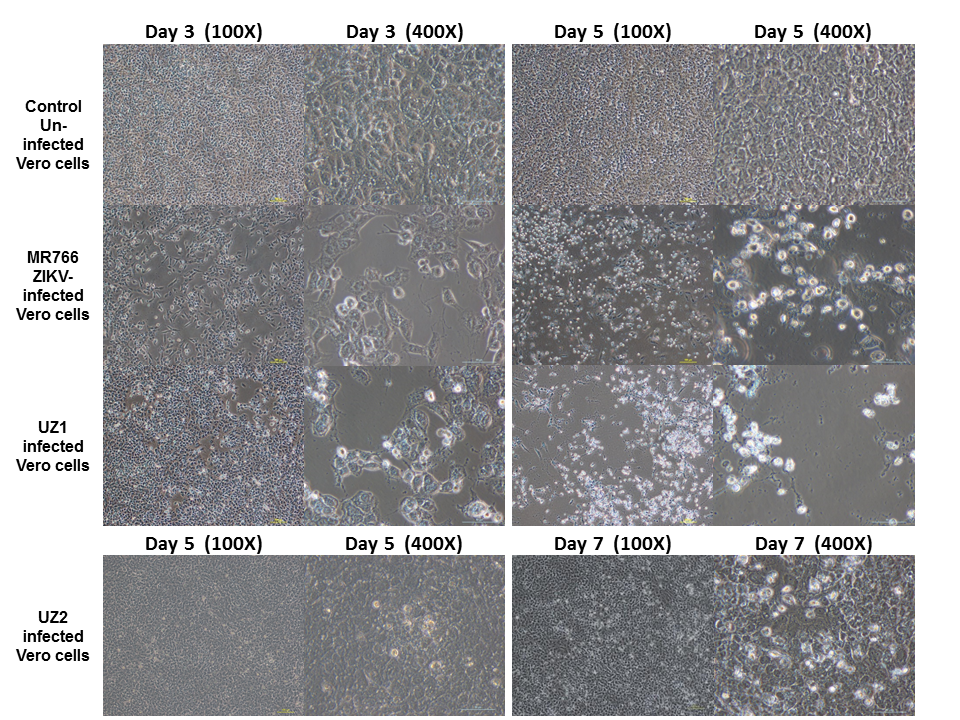

Supplement: S2 Fig — A1 (100X) and A2 (400X): Vero cells seeded in the TCID50 assay well inoculated with supernatant of the control U937 cell culture showed no CPE changes at day 3. Vero cells in the assay wells inoculated with the inoculum prototype MR 766 strain ZIKV and supernatant of the persistently ZIKV-infected U937_ZIKV-1 cell culture showed prominent CPE changes at day 3. B1 (100X) and B2 (400X): Vero cells seeded in the TCID50 assay well inoculated with supernatant of the control U937 cell culture showed no CPE-associated cytolysis at day 5. Vero cells in the assay wells inoculated with the inoculum prototype MR 766 strain ZIKV and UZ1 from supernatant of the persistently ZIKV-infected U937_ZIKV-1 cell culture showed prominent CPE with extensive cytolysis and cell sloughing at day 5. Vero cells in the assay wells inoculated with UZ2 from supernatant of the persistently ZIKV-infected U937_ZIKV-2 cell culture had clusters of cells with atypical CPE changes at day 5. However, no cytolysis or cell sloughing was seen in the well (A1 and A2). Vero cells in these TCID50 assay wells had no cytolysis or cell sloughing even at day 7 post inoculation of UZ2 from supernatant of the persistently ZIKV-infected U937_ZIKV-2 cell culture (B1 and B2). (TIF) [file pone.0203331.s002.tif]

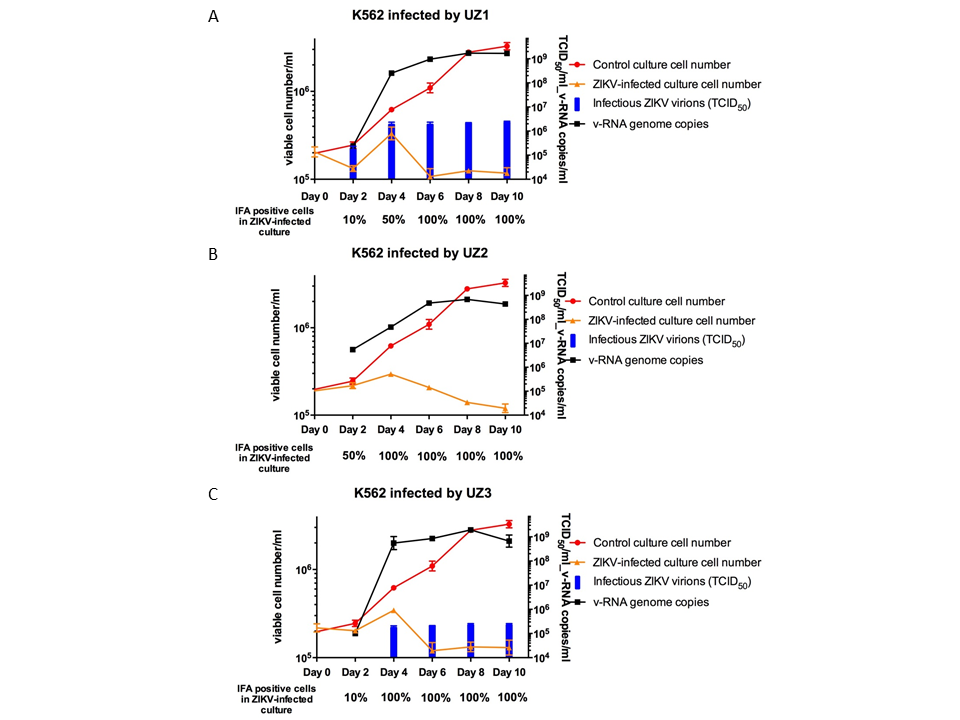

Supplement: S3 Fig — Cell growth, numbers of cells IFA-positive for ZIKV antigen, productions of ZIKV RNA genomes and infectious virions in cultures of K562 cells infected with the 3 persistent ZIKVs UZ1(A), UZ2 (B) and UZ3 (C). The cultures of K562 cells (2×10^5^ cells/ml) were infected with the 3 persistent ZIKVs (~107 copies of ZIKV v-RNA genome/ml) prepared from the culture supernatants of persistently ZIKV-infected U937 cell lines U937_1-ZIKV, U937_2-ZIKV and U937_3-ZIKV. Prominent CPE with extensive cytolysis and cell loss were seen in all the 3 cultures infected with the 3 persistent ZIKVs UZ1, UZ2 and UZ3. The amounts of ZIKV RNA genomes and infectious virions produced into supernatants of the ZIKV-infected cultures were quantified by qPCR and titrated by TCID50 assay against Vero cells. (TIF) [file pone.0203331.s003.TIF]
